# Part I. Spirit of the English Periodicals, and Notices of English Medical Literature

**Published:** 1834-01-01

**Authors:** 


### Periscope;

OR,

### CIRCUMSPECTIVE REVIEW.

"Ore trahit quodcunque potest, atque addit acervo."

I.

### Spirit of the English Periodicals, and Notices of English Medical Literature.

I. The Treatment of Asiatic Cholera and Chronic Diarrhœa, with Antimony. To which is appended Instructions for the Guidance of the Public, the most simple and efficient, to diminish its Mortality. By J. Langford, M.R.C.S., late Resident Surgeon and Superintendent of the Knott Mill Cholera Hospital, Manchester. 8vo. pp. 34, Ridgway, October, 1833.

When the cholera was raging at Manchester last year, Mr. Stott, of that place, shewed Mr. Langford a notice in the Medico-Chirurgical Review, of Dr. Reich's paper, published at Berlin; wherein Dr. R. details a very successful mode of treating cholera by tartrite of antimony. The practice was adopted by Mr. L. at the cholera hospital, and the present pamphlet is the result. Mr. Langford passes very slightly over the etiology and the pathology of the disease, of which, indeed, but little is known, in order that he may concentrate his remarks upon the treatment.

Our author divides the disease into three stages—in all of which he assumes the presence of serous evacuations—and arrested secretions, combined with the other usual symptoms of the malignant cholera, as necessary to constitute the Indian disease, so called.

"The first class or division—with *the skin* and tongue *warm,* and *tolerable* pulse.

The second class — the skin and tongue *cool* or icy cold, with *feeble* pulse.

And the third class—pulseless, and every symptom in an aggravated ratio."

By the numerical returns at the end of the pamphlet, we find that the first class was treated with great success by the antimonial plan, and without any consecutive fever. Amongst the second class were many patients of low dissolute habits and emaciated constitutions, labouring under organic diseases of old standing, with much less chance of recovery, in fact, than many in the third class. In the third class there were seven individuals who had bloody stools.

The plan of treatment which our author has followed is the exhibition of small and repeated doses of tartarized antimony, aided by *copious diluents,* till full, efficient, and continual vomiting is produced—not by one solitary effort, but by gentle, continual means.

"In proceeding with this stage of my communication, I shall confine myself to the explanation of that plan of treatment which I have proposed, and which upon a numerical return I found *decidedly* to give the most satisfactory result, more particularly in that distressing and difficult period, the pulseless collapse. This plan consists in administering small and repeated doses of *Tartarized Antimony,** aided by the

* "Dissolve ten grains of tartarized antimony in seven and a half ounces of distilled water, with half an ounce of rectified spirit, of which give half an ounce every two hours. Toast and most *copious dilution*. I order at least *half a pint* of toast and water, if preferred, or even common water, either tepid or cold, as may be most agreeable, to be given at one draught every *ten minutes or quarter of an hour*, to keep up full and *efficient* vomiting, taking care to avoid *ineffectual retching*. Some patients however have taken gallons in a few hours: no sooner is it swallowed, than it shortly returns, giving, as the patients *invariably express*, continual relief; as the gorged vessels of the venous system are for a time unloaded, and the sense of oppression at the epigastrium is diminished; and from the relief thus obtained, fluid is again and again demanded, affording us the opportunity of repeating this restorative process.

This continual operation of vomiting appears to me to be conducive to the following ends:—

To unload for a time the large internal vessels of the venous system, which during collapse are gorged with deteriorated blood, which blood is deprived of those functional powers usually attributed to its office. To call into action the diaphragm, by which the vitalizing influence of the respiratory functions are aroused. The heart by the same operation is unloaded of its vitiated fluid, and the vascular action is frequently increased to the extent of producing a pulsation at the wrist, which before was imperceptible. An immediate change will be observed in the fluid ejected, in which FLOCCULI ARE NO LONGER TO BE SEEN, and the *quantity* ejected, which before was copious and exhausting, is now diminished, not exceeding in quantity the amount administered, indeed less—*direct evidence of a specific change in the morbid action of the stomach.* This is an important fact.

This amended action, when produced, will be observed to continue its course through the whole alimentary canal, the stools becoming thicker or more gruelly, although from the greater extent in the intestinal surface, the dejected fluids will require a longer period to give the same evidence of their improved condition. So that a double action is observable in this stage (collapse) to be the result, viz. a continual mechanical action which contributes to overcome the torpor of the vascular system, and the atony of all the functions requisite for the restoration of the animal economy, equalizing the balance of the circulation, arousing the nervous energy; and, secondly, having a specific effect most probably on the mucous membrane of the alimentary canal, causing a diminution of the excessive exudation; permitting, through these media, the conservative principles of the constitution to rally against the morbid impression, under which the nervous system is rendered torpid; and, through that system, all the functional derangements appear to have their origin.

The very character of the vomiting is changed, it is no longer the *characteristic squirt*, which appears to be the sole effect of the stomach, but it assumes a general muscular action decidedly *remedial*."

In aid of these measures, our author has often applied, with advantage, cloths dipped in warm spirit of turpentine, over the thorax and abdomen, for the space of twenty minutes, and kept hot by towels. Frictions he considers as useless, or even prejudicial, exhausting the patient, without remedying the symptoms. The vomiting appears to relieve the cramps by diminishing the internal congestion, and more particularly, the author thinks, by allaying the morbid irritability of the intestinal canal. He continues the antimonial solution, every two hours, till the biliary and urinary secretions are restored. When bile is fully apparent in the ejected fluid, he gives an enema of gruel, salt, and oil, together with a small dose of castor oil by the mouth.

"As the various functions are restored from the torpor of collapse, I view the operation of the antimonial in a different light; the system is now disposed to run into an excess of action, and be destructive by consecutive fever.

---

water ad libitum. Give no other remedy."

May not the known powers of this medicine, by equalizing the circulation, now act upon a conservative principle, and thus avoid, as it does almost in toto, this consecutive stage? The remedy is by this time usually *tolerated* by the stomach; and the vomiting ceases.

I have seldom had to encounter consecutive fever; but in every case I have been enabled to arrest its progress. When there has been a long state of pulseless collapse, say for forty-eight hours, it is not to be wondered if there is some slight succeeding excess of action, even under this treatment. The usual *absence* of this consecutive stage, which is practically found as destructive as the stage of collapse, must give *considerable weight and importance* to this treatment."

The return of secretion giving proof of the system passing into another state, great care is necessary to save the *head*. If the antimonial was not sufficient, aided by enemata, our author immediately applied leeches to the head.

The following is the result of 94 cases treated on the antimonial plan. In 28 cases of class 1, all recovered—out of 36 cases of class 2, twenty-five recovered and eleven died—out of 30 cases, class 3, eleven recovered and nineteen died. Total—64 recoveries to 30 deaths. A letter from Mr. Ollier, and one from Mr. Stott, are appended, confirmatory of Mr. Langford's statements, and of the success of the antimonial treatment in their own hands. The following concise code of instructions, for the guidance of the non-professional public, concludes the brochure.

"No time should be lost in sending for medical aid.

This disease more frequently commences during the night, in a violent form, indicated by *vomiting* and *purging*, the severity of which is usually so overpowering, for the space of from one to four hours, as to bring the person immediately to a state of disease, too often both hopeless and irrecoverable. This form of disease cannot be mistaken.

I beg to press upon attention the high importance, and the great advantage of obviating the *loss of time*, which must pass, before aid can be had.

In nine cases out of ten, the patient has been labouring under the attack several hours, before medical aid is had recourse to, when the disease is found in *an advanced stage*.

The moment it is *suspected* to have appeared, by *vomiting and purging*, or either, *take one-fourth part*, or *two tablespoonfuls* of the following mixture, every *two hours*.

Tartarized Antimony, two and a half grains; Distilled Water, four ounces; Rectified Spirit, two drachms. This mixture to be kept ready in the house.

To aid *this vomiting*, drink half a pint of tepid water, every quarter of an hour, until medical aid arrives to direct its omission or continuance.

For children under *seven*, *half* the dose above named; and under *two* years of age, a *teaspoonful*. To be most particular in aiding the vomiting, by *draughts of tepid water*, or toast and water; if during the night, warm water cannot be had, drink cold.

By following these simple instructions, the prompt advantages derived, are, that an important remedial action is immediately produced; preserving the heat; relieving the cramps, if present; and checking the *excessive purging*, which otherwise would be going on; and too often, even *in one hour*, bring the person to that state, in which death makes sure of his victim.

I have generally averted the disease by this *efficient* and *simple* practice, if had recourse to *without* any *delay*, and restored the patient in a few hours. In others it has conducted to a favourable issue.

The *loss* of *life*, by this *early attention*, being *most insignificant*, disarming at once, this scourge of its dreadful mortality.

Again, I say, do *not permit* delay.

Use *no other* remedy; rigidly *abstain* from laudanum, brandy, and stimulants."

We confess we attach considerable importance to this pamphlet, since we had an early prepossession in favour of emetics in cholera, from observation of its effects in driving the blood to the surface, and relieving internal congestion. If the reader will turn to page 276, Vol. XVI. of this series, he will see that the Editor of this Journal proposed the plan of emetics, in a paper read at the Westminster Medical Society, on the 26th November, 1831. The 15th proposition begins thus: " The first internal remedy which I propose, both in aid and in imitation of Nature, is a stimulant emetic, &c." *ut supra*.

We recommend in the strongest terms, to our professional brethren, a full and fair trial of the plan proposed by Mr. Langford.

---

### II. NERVOUS AFFECTIONS OF VOLUNTARY MUSCLES.

Mr. ED. LEE, in a sensible little work on nervous disorders in general, has made some observations on those affecting voluntary motion. They depend, he remarks, on a state of excitement or atony of the faculty of volition; and may be induced by moral impressions or visceral irritation. In treating these affections, the patient's mind should be abstracted as much as possible from their complaints—mental or bodily irritation removed—and the functions of the chylopoietic organs improved, as far as in our power. We subjoin two short cases, from hospital practice.

*Case* 1.—" An unmarried female, æt. 20, was admitted into St. George's Hospital, in July, 1827, having two months previously fallen and hurt her left elbow and hip. Considerable pain and discoloration of the elbow were caused by the accident, but subsided after the employment of a liniment. When received into the hospital, the elbow-joint was in a state of semiflexion, and the fingers and thumb firmly closed. While the patient was awake, manual attempts to overcome the contraction causing a kind of hysteric paroxysm. She complained of pain extending from the elbow to the wrist; this was aggravated by moving the fore-arm, and by lightly pinching up or tapping the skin. The sensibility of the skin in other parts of the body was also morbidly increased, but her general health was not impaired. Mr. Brodie, whose patient she was, prescribed the application of the spirit lotion of the hospital to the elbow, and the following medicine: Tinct.: Valer.: Ammon.: Vini Alôes āā ʒj. sexta quâque horâ ex aquâ.

The patient feeling relieved by these means, they were continued, with the occasional employment of the shower-bath, for about a month; at the expiration of which period, the pain having entirely subsided, and the patient having regained the use of the elbow and hand, the contraction recurring only for a short time at distant intervals, she was placed on the out-patients' list."

The second case is extracted from Mr. Lee's notes, while attending the hospital at Florence.

" *Dec.* 10*th*, 1830.—Three months ago, a girl æt. 17, in whom menstruation was occasionally irregularly performed, but healthy in other respects, on descending into a close cellar, fainted, and fell to the ground. In falling, she struck her neck against some projecting body; abscess formed in the situation of the injury—was opened—and healed at the expiration of six weeks. Some days before her admission to the hospital she lost the use of her left arm, and shortly after, that of the left leg; the extremities of the right side subsequently became paralytic, and she was brought to the hospital in this state in the beginning of November. The intellect, the functions of respiration and digestion continued unimpaired, as did those of the detrusor urinæ and sphincter ani muscles. The case was considered to be inflammation of the spinal marrow. Repeated bleeding, the application of leeches and blisters along the spine, low diet, the exhibition of strychnine, and the formation of a sore by caustic in the situation of the previous abscess, produced no amelioration.

A fortnight ago she suddenly heard of the death of a near relation; and, from that time, constant movements of the limbs succeeded to the state of paralysis in which they had previously lain. These movements have continued ever since, the arms are incessantly beating against the breast, the thighs and legs alternately bent and extended with violence. Though pale, her countenance does not indicate the existence of organic disease, the intellectual and vital operations are not impaired, she answers questions readily, the tongue is clean, the pulse weak. The prognosis delivered by her physician is unfavourable. She takes no medicine, but leeches are occasionally applied along the spine.

*Dec. 24th.* The depletory measures have been discontinued, and the quantity of food increased, since the 14th. The patient has had, during the last two days, several hysterical symptoms, such as tremulous motions of the eyelids, loss of voice, occasional fits of laughter. The movements of the limbs are less violent, and at times cease altogether, she sleeps well, and her appetite is good.

*Dec. 30th.* The patient, having been allowed a more full diet, is much improved in appearance ; the movements are now almost entirely confined to the hands, and cease if her attention can be drawn off from her complaint."

Some cases are also given from Sir Charles Bell's Exposition, and other sources.

Mr. Lee's little work is of a practical nature, and worthy of attention, the author being a gentleman of observation, who has carefully studied his profession in this and in other countries.

---

### III. Compendium of Osteology; with an improved Method of preparing Bones. By Dr. Witt.

There is considerable originality, as well as ingenuity, in this Compendium. The following extract will shew the grounds on which Dr. Witt's peculiar mode of describing the bones is founded.

" In the following Tables an attempt has been made to convey a methodical knowledge of Osteology—the result of a practical mode of teaching this branch of anatomy. This mode consists in taking up each bone in succession, and placing and retaining it in one given and fixed position until the different parts presented to the eye—whether processes, foramina, or grooves—are *read off* in this lucid order and succession. The bone is then turned as upon a given axis, to bring into sight a fresh collection of parts. The advantage of this simple plan can scarcely be imagined without actual experiment.

In describing any bone care must be taken not to pass over a single point enumerated, whilst it is retained in its first position; and when every part shall have been described in that view, it then must be turned to its next aspect. Every thing depends upon this careful consecutive description; and when the knowledge of a bone shall have been acquired after this method, it will be really difficult for any part to escape notice, or fail to be impressed upon the memory; the eye forms a correct picture of the bone—the mind seizes the arrangement—and the memory retains the classified knowledge thus systematically acquired."—*Pref.*

How far this mode of description may be superior to that in common use, it is impossible for us, at present, to predict. Time alone can solve that problem. In the mean while, we have no doubt that any ocular auxiliary to the memory, that can be brought into operation, will be of great advantage to the student. The more senses that can be impressed, at the same moment, with any image, the longer the impression will last on the sensorium.

The mode of preparing bones for osteological purposes is conveyed in the following extract.

" About twelve years since my attention was particularly directed to this subject, by finding that some bones which I had macerated were unusually white, and free from smell. I continued for several subsequent years to macerate and prepare bones, as I conceived, precisely upon the same plan as that in which the maceration had been so successful; but although some proved tolerably white, the majority were cleaned with much difficulty, and when the ligamentous attachments were removed by dint of hard scraping, the bones were ever after yellow at the extremities, and had a more or less offensive smell. After much thought on the subject, I could not discover wherein my method differed from that in general use, but the preparation of another skeleton, about four years back, furnished me with materials whereupon to build something like a tangible theory; and this theory having been verified by repeated subsequent trials, I feel confident in recommending the practice founded upon it, although perhaps the reasons advanced may not be altogether conclusive. Two words, however, if properly understood, will furnish all the information that is necessary, viz. UNINTERRUPTED PUTREFACTION; for if the putrefactive process be in any way interrupted, the bones will never be clean. In order to obtain this end, the following directions must be scrupulously observed:—It is desirable to get the animal, of which a skeleton is to be made, as few hours after death as possible, while the blood is in an almost fluid state, and having taken off the muscles tolerably clean, and separated every bone, they should be immediately thrown into cold water; the water should be changed every twelve hours for three or four days, until it becomes no longer tinged with blood. A tub, or large earthenware vessel, must then be procured—if a tub, it must be well made to secure it from leaking during the long period required for maceration, and of such a size as to hold a sufficient quantity of water, over and above that which covers the bones, to allow for the waste by evaporation. Evaporation entails two difficulties, for if it go so far as to leave the ends of some of the longer bones projecting out of the water, they immediately become quite black, and if fresh water be added to cover them, the whole putrefactive process is arrested: hence the vessel must stand in some covered out-house, secure from the admission of rain, and from the danger of the water being drunk by rats. So far as I have observed, the vessels should be merely lightly covered over, as a certain access of air appears necessary—for, on one occasion, being without a convenient place, I buried some tubs during the usual period, when the bones proved the most offensive that I ever prepared, and it was an endless task to get off the ligaments.

Should all go on successfully, and the process be in no way interrupted, either by evaporation or by leakage, in the space of about six months, the bones may be washed perfectly clean with a common brush, the ligaments and muscular attachments may be pushed off like a cake from the ends of the bones, and then if they be held up, a thick fluid will be seen to exude through every aperture from the interior, proving that the putrefactive process has gone on in the interior, with the same happy result as on the exterior. It is hardly necessary to observe, that the internal and external putrefaction must go hand in hand in order to procure a clean bone, and this I apprehend to be the general source of difficulty. A vertical section of any of the cylindrical bones in my collection exhibits the interior even whiter than the exterior; and the cancellous structure is a beautiful white net-work, unsoiled by any medullary matter. After the bones have been well brushed, they should be soaked in clean water twenty-four hours, and then carefully cleaned with a scalpel from all ligamentous and cartilaginous matter that may be found still adhering, but the bone should in no way be scraped more than is absolutely necessary, as it is deprived of all its minute processes and distinctive characteristics. The long cylindrical bones should be placed upright upon their extremities for a short time, in order to allow of the entire escape of the medullary fluid. To cleanse them from any oleaginous matter, whether external or internal, I have generally soaked them for the next forty-eight hours in a solution of subcarbonate of potass, in the proportion of about a pound to each gallon of water, after which, they should again be well washed and left for a short time in a large quantity of clean water, and then wiped with a dry cloth. The bones after this should be carefully laid upon a clean deal board, and exposed for a few days and nights in the open air, taking the precaution to turn them now and then.

As to the exact period for maceration, six months has been stated as a general time; but this is found to vary, as a set of bones will macerate in almost half the time during the summer months, to what will be required in the winter. The bones of small animals, and of birds also, require a comparatively short period; and it may be observed that the bones of the ruminating class of animals always macerate more speedily than those of the carnivorous."

---

### IV.

### THE PRECEPTOR,

### DIDACTIC AND CLINICAL.

### No. I.

The publication of prelections, at least during the life of the lecturer, and especially during the delivery of the lectures themselves, forms a kind of epoch in medical literature; and the introduction of such a procedure is unquestionably due to our indefatigable and bold contemporary—the Lancet. At first the practice gave rise to warm discussions, both moral and legal—and the longest head in the law—that of Lord Eldon, was not a little puzzled and perplexed as to the justice—or rather the legality of the proceeding! The knotty point was, at length, settled in a true forensic style. If a lecturer had a good memory, and could deliver his lecture off hand, without book, he was lawful game, and his prelections might be published the next day at Charing Cross, or in any other way that the note-taker chose. But if the lecturer had a treacherous memory, and was obliged to write down his lectures, as some preachers do their sermons, he was under the protection of the Court of Chancery, and it was unlawful to publish his lectures! There is a nice point of distinction, both in law and justice, for you!!

There is now, however, no question about the legality or the morality of the thing. So soon as teachers perceived that there was more gain than loss by the publication of their lectures, the question of *right* was waived, and the publisher was not merely permitted—but assisted in the publication. This being the case, and most of the lectures being either furnished or revised by the teachers themselves, their doctrines and practices become just as legitimate objects of criticism or review, as any regularly-printed work, presented to the public through the usual channels.

Medical lectures, are, or ought to be, a compendious and lucid enunciation of those doctrines and practices which are most generally adopted by the most experienced part of the profession—and not of the peculiar opinions or modes of treatment embraced by individual lecturers. The latter should be mere sprinklings—and the more they engross the body of the lectures, the worse it is for the pupils, when they come afterwards to put the precepts of the schools into practice. This eclectic mode of tuition, however, is not the most attractive, either for the student or the public—and, therefore, it is every day more and more neglected—we might say avoided. It is not unusual to see a whole course of lectures, consisting of a scarcely-interrupted string of egotisms—of what "*I* do—what *I* think—and what *I* would recommend." This is a much easier mode of tuition, than a comprehensive exposition of what the most experienced practitioners do, think, and recommend generally. It is much more acceptable, too, to the pupil—who goes away an echo to the ego; proud and happy that he has only to put his preceptor's edicts into execution, to become as distinguished as the prototype! Teachers well know the propensity of neophytes, "jurare in verbo magistri"—and he must be an inobservant or very limited practitioner, who does not every day trace the master in the man—the school, rather than the science, in application to actual practice. The consequences are, contracted views, disappointments, and embarrassments from want of resources. Were it not a violation of private confidence and honourable secrecy, we could adduce the most striking examples of the mischief which results from the attempts of the young practitioner to wield the *extraordinary* arms of his master. *Heroic* remedies are not safely administered—except by Heroes:—and even these last sometimes cure more diseases in the lecture-room, than in the sick chamber. The great difficulty, in practice, is to know the *when* and the *where*, to employ active remedies:—the great object of the student, on the other hand, is to arm himself with the quo, rather than study the quomodo. It is not nearly so much to the inefficacy of medicine that we owe failures, as to the misapplication of it.

Lectures are of much greater consequence than books—because they are delivered to those who are incapable of judging what is right from what is wrong—whereas books are addressed to practitioners, who are or ought to be, capable of judging of their merits by the sure test of experience.

Under all these circumstances, we conceive that published prelections are legitimate subjects for criticism, and that they come fairly under the *review* of the periodical press. They are not, however, subjects for analysis, because like *books of elementary instruction*, nine tenths of them are or ought to be matters as familiar as A. B. C. to practitioners. It is only, as we said before, to peculiarities, or personal opinions and practices that our attention will be directed. Neither shall we attempt to notice *all* the lectures delivered in this great metropolis. Some of them are too good—and require no critical notice—others are too bad, and incapable of correction. It will be chiefly to clinical lectures that our critical notices shall extend. In this first paper, however, we shall only notice a few of the introductories of the present medical session. In other parts of our Periscope, other lectures will be reviewed.

### The Introductories.

1. Dr. Grant. Among the introductories of this season, that of Dr. Grant, at the London University, claims especial notice. Its facts are divested of verbiage—and its counsels are full of wisdom. It is free from boasting vanity—and indulges little in visionary anticipations of unreasonable prospects and success. Dr. Grant briefly alludes to the economy introduced into all the arrangements of the University, and dwells, with reason, on the advantages which must follow the erection of a clinical hospital. The application to Government for a charter and power to grant degrees, is said to have been favourably received, in high quarters; but we greatly doubt whether the University ought to, or need look to any other auxiliary than its own celebrity, for attraction. That celebrity must be the result of talent and industry.

The College of Physicians is truly represented as not only void of all utility to the profession at large; but as injurious, unjust, and oppressive. "The object of such a College is absurdly perverted, and very few of the eminent physicians of England can obtain a fellowship, or expect to enjoy those advantages and privileges which are attached to it." It is anticipated by Dr. Grant, that a scrutinizing legislature will retain whatever is good in the old institutions, and correct those abuses which have only the sanction of custom and antiquity. The advice to students, in respect to their studies and conduct generally, is judicious and quite unobjectionable. We shall conclude this notice with the following biting, but just reflexion on the system of apprenticeship—a system against which we have always waged war.

"The system of apprenticeships to apothecaries and surgeons is a system of menial occupation, idleness, or vice; it is a remnant of the low and ignorant state of the profession in olden times; it serves only to secure pecuniary advantages and gratuitous service to a few interested men, but is ruinous to the education and character of our youths, degrading to the medical profession, and injurious to the community at large."

2. Dr. Watson. Dr. W.'s introduction this year, is a temperate and judicious appeal to the best feelings and best interests of the students. While he gives due credit to the exertions of the Apothecaries' Company in raising the scale of education among the great class of General Practitioners, he does not conceal his disapprobation of the apprenticeship system. He urges a regulation that may abridge the indenture and enlarge the curricula of the schools. On all branches of elementary medical education, Dr. Watson's advice is sound. On medical politics, beyond the allusion to apprenticeship, Dr. Watson does not touch. The leaden sceptre of the College, dark and heavy as the sooty columns of the portico, hangs over the head of every Fellow, and threatens to descend like the guillotine, and cut short any sentiment of reform not congenial with the despotism of the temple in Pall Mall East! The spear of the Cossack and the bayonet of the Croat, do not more effectually quench the fire of liberty on the plains of Poland and Lombardy, than does the mace of our Eighth Henry, put down every expression of liberality in a body of men who ought to take the lead in medical literature and science!

3. Mr. Wardrop. It is not stated where Mr. W.'s introductory lecture was delivered; but it appears to be rather an introductory preface to a series of surgical essays, than a viva voce lecture to a class. Be this as it may, the address turns almost entirely on *Order* and Classification. We are glad to have Mr. Wardrop's authority for "Order being a faculty of the mind," exemplified by the child, "who begins to select and arrange such material objects as happen to be within his reach." Mr. W. has a large family, and therefore must have had ample means of observing the propensities of children. For our own parts, we have not been so fortunate as to have the *faculty* of order very early developed in our own family. On the contrary, the *disorder* which our children have almost daily produced among our books and papers, has tended very much to weaken our faith in the phrenological location of this faculty. We are, indeed, strongly inclined to look upon Order, as the offspring of reasoning and reflection; founded on observation of its utility, rather than on any instinctive or innate propensity of the mind brought into the world with us. But whether innate or acquired, we highly respect the faculty, and recommend it as strongly to our younger brethren, as Mr. Wardrop can do. The introductory lecture or preface, in question, is deserving of the pupil's careful perusal.

4. Mr. Guthrie. We ought not to pass over the introductory lecture delivered by this able and eloquent teacher, at the Medical School, Little Windmill Street, on the 2d of October. Mr. Guthrie's medico-political character has, to our knowledge, been greatly misrepresented, and, consequently misunderstood by those who were not personally acquainted with him. He may be a Tory in general politics; but every man has a right to embrace whatever political system he pleases. We know that in medical politics he is a Reformer, and that is enough for us. No man has laboured so hard in the work of reformation at the College of Surgeons as Mr. Guthrie. He has tried to effect reform, when some of those who bellowed loud for it out of doors, did not lend their aid in the Council-room! Mr. Guthrie may be personally vain—with that we have nothing to do. He certainly has better reason for being vain than some whom we could single out, and who are proud enough! Mr. G. avers, in his introductory lecture, that he is only proud of two things—1st. Of never having written anonymous letters or papers reflecting on the character of another—and, 2ndly. Of never saying that behind a man's back, which he would not say before his face.

Mr. G. corrects at length some misstatements that have been made respecting his negociations with the late Mr. Brookes for the latter gentleman's museum. Mr. Brookes was a wrong-headed man, as far as his pecuniary matters were concerned; and had he followed the advice, or taken the counsel of Mr. Guthrie, he would not have died in poverty. It is very unfair to attack Mr. Guthrie for the false judgment or imprudent actions of Mr. Brookes. Besides, Mr. Guthrie was, at the time alluded to, only a junior member of the Council, and consequently had but little influence among the antiquated aristocracy of Lincoln's-Inn-Fields.

Mr. G. declares, and we can bear witness to the truth of his declaration, that so far from endeavouring to support abuses in the College, and maintain the existence of those laws that are incompatible with reform—he is, in these respects, neither a Tory nor a Conservative, but a "regular Radical." It was to him mainly that provincial schools owed the privileges which they now enjoy. Mr. G. was, in fact, denominated "*their* Joseph Hume," within the walls of the College! A charge of neglect at the Westminster Hospital has been made against Mr. Guthrie—though it is well known that he pays more attention to his hospital duties than almost any surgeon in London—whenever extra-attention is necessary.

It is well known that, in pecuniary matters, no teacher in London is more liberal than Mr. Guthrie—a character, indeed, which most medical men who have been in the service of their country during the late war, have brought into private life, in the exercise of their profession. One of his pupils has published a defamatory statement respecting Mr. Guthrie, which has been keenly commented on by the teacher. Mr. Guthrie's lecture-room has never been closed against pupils who were unable to pay the fee—and it has often happened that pupils have attended a whole course, without ever asking Mr. G. for permission, and then come to him for a certificate! This is not a little surprising; but it is still more surprising that Mr. G. has often given the certificate, under the above circumstances, when assured that the pupil's attendance had been regular, though furtive!

After all this, which is nothing but the truth, we cannot let Mr. G. off, without some censure. In the following address to his pupils, last year, the teacher is perhaps rather too cavalier. Mr. G. has relied on an honest consciousness of independence and superior knowledge.

"You are entitled to sixty lectures and no more, they will be on what subject I please and as I please, and I will not lecture in May, even if the course be only half completed. If you do not like this, gentlemen, do not come to me. I do not wish you to do so unless you are satisfied it will be for your advantage; and clearly understand, that you are entitled only to what I please to give you."

Mr. Guthrie, however, is much more inclined to "speak daggers" than to use them, as will be seen from the next passage.

"Now, then, what did I do from Christmas to May? I lectured three, four, and five times a-week, giving more than eighty lectures instead of the promised sixty; and in the month of April to my own very great inconvenience. When the last day came, I said, 'We have been obliged to hurry over the last two or three subjects, and the diseases of the eye have not been noticed. Come to the Ophthalmic Hospital every Friday, see all that is to be seen; I will make some clinical remarks to you afterwards, whenever I have time, and you will, I hope, reap a greater advantage than you could do from the delivery of a few dry lectures here without the opportunity of seeing the diseases themselves.' For all this trouble and kindness one of you has thought proper to write a letter abusing me in the Lancet, and to do it at the commencement of this session, warning students not to come to my lectures; I am afraid he must be one of the gentlemen who have some crosses against their names in my list for absence when it was called over, but whether it be so or not let us understand each other. If any one believes I lecture here for gain in money it is a mistake. The class last year consisted of one hundred persons; sixty paid, the remainder belonging to the army, navy, old pupils, &c. did not. Of the sum received about 120 pounds came to me, after paying all expenses. When solicited to lecture at other places, where I might perhaps have got three times as much, I have always refused. I would not go one mile further from home for the money; I would not expose my servants and horses to the cold for it, it is no object to me. The favour of the public, much I am willing to acknowledge above my desert, which has given me a large and annually increasing income, has rendered it unnecessary. I lecture, then, gentlemen, upon principle. I owe to the medical department of the army a great debt of obligation. It is to them I am indebted for the situation I hold in this metropolis; to those who served with me, and who supported me afterwards. It is little I can do for them, but I can assist their successors; when they want information I can obtain it for them; when they wish to renew any part of their knowledge I can assist them, and instead of being sneered at, as they often were, some 20 or 30 years ago, for some trifling defect, by men who did not know them, they, and all other officers in the public service, find in me a friend, I wish I could say as capable as he is willing to befriend them. As Surgeon to the Westminster and the Royal Westminster Ophthalmic Hospitals I think it right, whilst I have health and strength, to give some public instruction, until younger men shall arise in each capable of taking my place, when it will be most readily resigned. So far from money being my object, you all know that the door of my lecture room is never closed. That no one is ever asked for his ticket or his name. You know that some gentlemen have regularly attended for a whole season without one single word being said to them, and some have even tried my good nature so far as to ask me for a certificate at the end of it, even without paying the fee. I have never refused it after being satisfied, on inquiry, that they had duly attended."*

So much for the introductories. There were many others delivered last October, of no doubt an interesting nature; but we are unable to include them here. In succeeding Numbers of the "PRECEPTOR," we shall revert to the subject of lectures generally.

---

### V. CYNANCHE LARYNGEA.

Under this head, Mr. J. Hey Robertson has made some observations, and detailed a case which we shall briefly notice. This inflammation is certainly the most dangerous of all the phlegmasiæ, not even excepting carditis or meningitis. The dyspnœa is so distressing, that it ultimately amounts to suffocation, if not relieved. The recorded cases shew a lamentable want of success, even where laryngotomy is performed. Bleeding is our sheet anchor, no doubt; but it often fails. Our author *decidedly condemns it.* This is going rather too far, we suspect. "From the œdema existing (says he), I should be inclined to infer an atonic state of the vessels of the part. We do not bleed with the hope of removing this elsewhere. To bleed, in effusion of the brain, is to produce more effusion." Now the state of the parts in laryngitis, is almost precisely the same as when boiling water has been taken in mistake. The œdema is the effect of inflammation; and although we may bleed too late in laryngitis, this is no reason why we should not bleed at all. When the effusion has gone to a certain extent, and suffocation is imminent, we fear that nothing but tracheotomy will save life. Mr. Robertson, however, has proposed a less herculean remedy—namely, the nitrate of silver, in a strong solution—40 to 60 grains in the ounce of water—applied freely by means of a small brush to the posterior fauces—between the arches—and as far down

---

* London Medical and Surgical Journal, No. 89.

on the posterior of the throat, behind the uvula, as possible; but taking care that the solution do not reach the epiglottis.

Mr. R. relates the case of a young lady of very delicate constitution, who had neglected an inflammation of this kind, till the symptoms were very alarming. Only two alternatives presented themselves to Mr. R.—to arrest the mischief locally—or open the trachea. He immediately applied a solution of the nitrate (60 grains to the ounce) freely to the parts above-mentioned, which required three applications of the pencil. After the second application, she recovered her voice. On the third application, some of the solution touched the epiglottis, followed by the most distressing efforts to cough and vomit. These subsided, and the recovery was rapid.

We see no objection to this remedy where the disease has got head, with or without bleeding.—*Glasgow Journal, No.* IV. *N. Series.*

---

### VI. Uterine Hæmorrhage, from Placental Presentation. By Dr. J. Maxwell.

In our respected Glasgow cotemporary for October last, an interesting case of this kind is very fully detailed by Dr. Maxwell. It furnishes an example in which the os uteri remained undilated, notwithstanding uterine contractions had been frequent during 24 hours—and hæmorrhage had gone on for 36 hours, to the great hazard of the patient's life. Dr. M. observes that this case proves that there are exceptions to the general rule laid down by obstetric practitioners, namely—never to force the hand into the uterus while its orifice is rigid. We shall now proceed to abridge the case very considerably.

*Case.* On the 25th of 3d month (we wish these Quakers would compute time like Christian people), Dr. M. saw his patient, who had been flooding since the preceding day, followed by labour-pains. The uterine contractions were easily recognized by the hand—but the patient herself was something unusual in the condition of the parts, as her pains had not the effect of carrying down the uterus. On trying a pain, no part of the uterus could be felt—but with the hand in the vagina, the os tincæ was found to be firm, and of a rounded figure—the cervix uteri by no means distended. A finger passed through the os uteri did not recognize any part of the child, nor of the placenta. The hæmorrhage was considerable. The tampon and other means were used. The hæmorrhage, however, continued, together with the pains, but without dilatation of the os tincæ, and the painful alternative presented itself, of seeing the patient die exhausted, or of attempting the delivery under circumstances the most unpromising. In company with a medical friend, the delivery was determined on. A decoction of the secale cornutum was prepared, and a glass of wine and seventy drops of laudanum were given. With immense difficulty, the hand was partially got within the os tincæ, when the placenta was discovered, lining that part of the uterus immediately above the contracted part. The fingers were pushed through it; and the liquor amnii was discharged. The feet of the child were seized, and easily brought down. The secale cornutum was now given, and the hand was withdrawn with considerable difficulty. Traction by the feet delivered the child, and the discharge afterwards was moderate. She had a good recovery. Many judicious remarks, and a reference to several analogous cases, are made by the author of the paper, for which we must refer to the journal itself.

---

### VII. Retroversio Uteri.

Mr. Cunningham has related a case of this kind, in the October Number of the Glasgow Medical Journal, of which we shall here condense the chief features.

*Case.* Mrs. B. 13th July, 1833, stated that, for ten days previously, she had been afflicted with pains in the abdomen, extending down the thighs, with frequent and painful micturition —costive bowels, &c. She considered herself in the fourth month of pregnancy with her second child. Venesection, calomel and opium—cathartics.

*Monday*, 14*th*. "I was hastily summoned to her bed-side; she had passed a dreadful night, with a constant bearing-down pain, and desire to evacuate the bowels, coming on in paroxysms, resembling the last expulsive efforts of parturition—the medicine exhibited the previous day had been ejected, and the vomiting continued the greater part of the night. I now began to fear that the real cause of these violent symptoms had not as yet been ascertained, and to satisfy myself whether abortion was not a threatened event, requested a vaginal examination. Immediately on introducing the finger, it was met by a large firm ball resembling the head of a full-grown fœtus. I was for a while puzzled what to think: from the early month of utero-gestation, even granting that abortion was being effected, the fœtal head must have been much smaller than the tumour I now felt. This opinion was therefore instantly discarded. Polypi—morbid structure —impacted fæces vaguely flitted through my mind, and for the moment I could come to no satisfactory conclusion. I then began to search for the os uteri, but it was nowhere to be found. On examining per rectum, the same globular ball was felt, but no fæces. An attempt to press the tumour up gave considerable pain, but had the effect of permitting the poor woman to void a considerable quantity of urine. Another fruitless search in quest of the os uteri determined me that this must be a case of retroversio uteri, and the first that had occurred in my practice.

On inquiry, she admitted that her former attendant (who, by the way, was not a regular licentiate) had submitted her to a similar examination, and only recommended the continuance of purgatives, which were regularly vomited, and the application of a blister to abdomen!

This woman's condition had now become exceedingly alarming; her strength was much exhausted, and unless something effective were speedily adopted, she would undoubtedly sink. It became a grave matter of consideration what line of procedure to adopt. The long existence of severe inflammatory action rendered it highly probable that important adhesions had taken place, which would render attempts at replacement dangerous. I could not, however, satisfy myself that any measure short of this end would be productive of benefit. At this juncture I availed myself of the opinion of Dr. Davidson, who, after examination, agreed as to the nature of the malady, and also thought that replacement ought to be cautiously attempted. Before proceeding to act upon this determination, I introduced the catheter with much difficulty, and drew off about a pint of very turbid urine. I again endeavoured to get at the os uteri, and persuaded myself that I could feel its posterior lip high up over the simphisis pubis, but found it impossible to make it available in the operation, as had been done by Dr. Weir in a similar case.* Contenting myself with introducing the fingers of the right hand, I pressed them against the body of the uterus gently, but steadily, till it sensibly began to yield; withdrawing them, I now got the forefinger of the same hand attached to the os tincæ, and tracted with it, while the thumb pressed against the body of the tumour, and thus with little difficulty, and almost no suffering to the patient, I succeeded completely in bringing the uterus into its original position. Although much exhausted and enfeebled, the woman expressed herself gratified at the accomplishment of our object, and thought herself relieved. A clyster was given through the course of the day, which produced some evacuation—the calomel and opium were continued in small doses, and the urine regularly drawn off.

Tuesday and Wednesday passed in a tolerably easy state, but the pulse never

* Glasgow Medical Journal, Vol. I.

decreased in number, ranging about 100, and feeble. The bowels began to act without medicine.

Thursday an increase of abdominal pain took place, which was combated by a small bleeding, and the application of turpentine cloths.

*Friday.*—Parturient pains were established, and the fœtus expelled after two or three hours' illness—placental separation tedious, but eventually brought away entire. There was little or no hæmorrhage, but the patient was now excessively exhausted, with a quick weak pulse, and hot skin. Much swelling of vulva, which remained till death.

Continued free from pain till the morning of Saturday, when she suffered another return of pain—the belly was now much increased in size, and so tender that it could not be touched, the pulse weak and fluttering. On account of her debilitated sinking state, my only resource was the application of a blister. Anticipations of success, however, were so small, that I was not surprised at next visit to find an aggravation of symptoms. Vomiting was now incessant—the matter having the appearance of coffee-grounds—the little lochial discharge which continued had a very offensive odour—the belly continued to swell; flushings—hiccup—and other deadly symptoms, betokened an early termination to her sufferings.

On Sunday she expired, six days after replacement, and four after abortion.

As this was a case replete with interest throughout, and one that had engrossed much of my attention, I was exceedingly anxious to ascertain, by a post-mortem examination, the pathological state of matters. With much difficulty leave was granted—and 30 hours after death, in the presence of Dr. Davidson, we proceeded to the

*Autopsy.*—On making a section through the abdominal muscles, the omentum and peritoneal coat of intestines were found highly vascular. On carrying the section down towards the insertion of the recti muscles, and over the situation of the bladder, a quantity of urine escaped from a wound made by the scalpel. On examination, it was found that this organ had become distended, and formed extensive and firm adhesions to peritoneum lining the muscles of abdomen. About a pound of effused fluid, with large masses of coagulable lymph, was found in abdominal cavity. The uterus was *in situ,* and contracted to its natural size; its mouth dilated and flaccid. This was the only cavity examined."

There can be little doubt that peritoneal inflammation was here the cause of death. It is probable that a more early rectification of the malposition might have saved life. The case is important, and deserves reflection.

---

### VIII. DISEASES OF THE POOR IN GLASGOW, FROM THE 16TH MAY TO THE 16TH AUGUST, 1833. By Mr. J. A. EASTON.

There were upwards of a thousand patients attended by Mr. E. during the above trimestral period, of which the mortality was 1 in 22. The prevalence of fever, dysentery, and influenza contributed to swell the list beyond the usual range. It is curious that the fatality was greater among those who were well fed and clothed, than among the most abject of the poor. Only one case of Asiatic cholera occurred, and Mr. E. details the case, and the circumstances attending it, with some minuteness, as proving the spontaneous origin of the disease. The individual was a female, who had been working harder than usual on the preceding day (29th of May), and went to bed well, but tired. At six o'clock the next morning, she was seized with diarrhœa, at first feculent, but afterwards watery. When seen at eleven o'clock, she exhibited all the features of decided cholera. By active means, chiefly by calomel in large quantities, she was saved. There was no other case before or afterwards in the neighbourhood, and, consequently, the idea of contagion was out of the question. Mr. E. concludes, and very justly, that all cases arise spontaneously, and are not propagated by personal contact.

The question will not be long agitated, since the common sense of mankind is beginning to prevail over prejudice and self-interest.

*Dysentery* has been severe among the poor. Our author's practice latterly consisted in the exhibition of opium alone, with occasional doses of castor oil—and was eminently successful. In some cases, however, it was found necessary to give calomel.

*Hæmatemesis.* This was chiefly of the passive character. Oil of turpentine, in doses of 20 drops every hour, succeeded in checking *this* kind of hæmorrhage.

Croton oil was extensively used as a counter-irritant in cynanche, laryngitis, &c. Three or four drops, he observes, are enough to bring out a papular eruption in less time, and with less irritation, than when tartar-emetic is used. But the query is, will these easy and comfortable eruptions prove as efficacious as the painful ones from antimony? We apprehend not. The very pain and irritation are the chief, if not the only causes of the consequent relief of symptoms.

---

### IX. Dr. Davis on Sterility.

[Fasciculus XXIII.]

Dr. Davis commences this fasciculus by remarking, that among the least known causes of sterility are certain changes and certain states, attendant on age, on constitution, or on habit. When we cite the instances brought forward by the Doctor, it will probably appear that the epithet "least known" is sufficiently deserved. Catherine de Medicis, Queen of Henry the Second of France, had been married for ten years before she gave her husband any promise of an issue; in the sequel, she blessed him with a numerous family. Anne of Austria was sterile for twenty-two years, and afterwards gave birth to the Grand Monarque—to Louis the Fourteenth.

When we stoop from these illustrious instances of barrenness, to contemplate the malady in those humbler females, whose condition allows the eye of scientific curiosity to pry more closely into the particulars, we find that the mystery is still unsolved. Dr. Davis relates, in his prolix style, the case which we shall now abridge.

A lady, aged 24, of delicate constitution and sanguineous temperament, became the mother of her first child in the sixteenth month of her marriage. The labour was difficult, and the convalescence tedious. She endeavoured perseveringly to suckle her child; but the attempt was injudicious, and she suddenly became subject to paralysis of the muscles of the left side of her face. The suckling was abandoned—the patient went to Buxton—and, after remaining for about nine weeks, she returned to her family perfectly recovered. In a few weeks she menstruated properly, and the function was afterwards performed with regularity. But for five years no conception ensued. At the end of this period, her only child experienced a dangerous accident, and the intelligence of his freedom from danger made her, to use her own expression, "almost wild with pleasure." In this tumult of the passions, the lady was exposed to the chance of impregnation, and conception was the consequence. She afterwards gave birth to six other children.

Other cases are related, in which the cause of the sterility and the conception would seem to be equally obscure. Mauriceau attended a woman, thirty-three years of age, whose pregnancy occurred nine years after marriage, and who never had another child. The disbeliever of the modest and the patient virtues of the female sex has been tempted, on similar occasions, to indulge a doubt. He has hinted that some other stimulus has been employed, than that to which the uterus of the patient has been accustomed. The scientific and experienced physician, acknowledging the fallacious uncertainty of knowledge, has felt compelled to admit his ignorance, and record the facts without an observation.

In other instances, some obvious changes in her habits, and probably in her physical condition, has enabled the female to conceive. Mauriceau cites a case of sterility apparently of this description.

A female was barren for fifteen years. She displayed no obvious ailment for the first twelve, but during the last three years she suffered from a complaint which reduced her to a condition of extreme debility. She visited the waters of Vichy in the Spring and again in the Autumn, and drank that celebrated tonic and aperient. Her health was surprisingly improved, and in four months afterwards conception followed.

Constitutional diseases frequently prevent the occurrence of impregnation. But this is not always the case, and Capuron has even reported that women have sometimes conceived and become pregnant during paroxysms of hysteria, syncope, lethargy, and seeming suspension of all the functions of life. Venereal excesses are commonly observed to interfere with the process of conception. The public prostitutes are a striking and a very familiar example of the truth of this remark. It is probable that many of this miserable class use measures to prevent or arrest impregnation. But making all allowance for what vice, ingenuity, and necessity will attempt, it must still be admitted that insulted nature is the powerful and universal agent in occasioning the barrenness of courtezans. The abuse of spirituous liquors is also a probable and extensive cause.

The fecundity of women is influenced on a great scale by climate. Fodéré has made some remarks on this subject, which Dr. Davis has transcribed, and which we shall transcribe again.

"The human race," says M. Fodéré, "is also, doubtless, the subject of favourable conditions in respect to its existence and its means of multiplication. A humid warmth of climate is that which would appear most to suit it, not so much, indeed, as a means of long life, but as a condition of its easy and rapid propagation. The extremes of heat and cold, and of dryness and humidity, are conditions of climates less favourable to the multiplication of our species. Lower Egypt has, at all periods, been represented as an immense nursery both for the human species and of animals. The same prodigious activity of the function of reproduction appears to extend along all the great rivers of Africa. The sea-coasts, both of the Ocean and of the Mediterranean, are extremely densely populated; a circumstance as much probably to be ascribed to the sweetness of the climate as to the habit of living upon fish, of which the meat is nutritious and easy of digestion, to which the inhabitants of those countries are addicted. Higher Egypt, on the other hand, the arid regions of the interior of Africa and Arabia, and all those countries which approach the arctic pole, and which stretch beyond the sixtieth degree of latitude, are less numerously peopled. In the province of Nice, after having witnessed the greatest fecundity in the basin which surrounds that town, and which forms its immediate territory, as also that of the valley of Nervia, we are surprised on ascending the heights of Perinaldo, to observe what a great number we meet with of young women who have never menstruated, and of married women who have never had families. I have likewise had occasion to make similar observations at Beuil, a district northward of the same plains. Both of these communes have their localities on very dry and elevated tracts of country; the one however having the advantage of a warm and genial aspect, whilst the other is exposed to one of an icy coldness. Again, whilst practising my profession at Martique, a neighbourhood peopled by fishermen and sailors, and remarkable for its swarms of children, I was often consulted by the inhabitants of Cape Couronne, which was not more than two leagues from Martique, for amenorrhœa and sterility. Now the elevated platform of Cape Couronne is precisely similarly situated in respect to its climate with the heights of Perinaldo." Fodéré, Pathologie et Médecine légale de la stérileté, Dict. des Scienc. Médic. p. 517.

It may be doubted if M. Fodéré has expressed the whole truth. Much as climate influences directly the fecundity of animals, its indirect action is perhaps the most important. The diluvial plains, the valleys, and the coasts that display their swarms of living animals, are the spots where food is most readily procured. The desert or the mountain would fail to sustain a numerous population, should such be permitted to arise. M. Fodéré may answer that this is not all, and may plainly urge that amenorrhœa and sterility are witnessed in the females that tenant these localities. But the want, or, at least, the deficiency of food not only prevents the generation of animals, but exerts a twofold influence on those who actually exist. Marriages and prolific connections are avoided, from the consciousness of the parties that misery and want must await their offspring and themselves. This moral evil, sufficient to account for those uterine affections to which M. Fodéré has alluded, is assisted in its operation by the physical debility that the want of the comforts and conveniences of life entails. On the whole the reflecting physician is surprised, that the operation of these causes is so feeble. It may reasonably be questioned, if the obvious and afflicting agency of cold, and hunger, and distress, is so unfavourable to the perpetuation of the species, as the evils at the opposite extreme, fashionable luxury and dissipation. The sturdy family of the Irish vagrant may admit of comparison with the sickly and the puling progeny of the debauched aristocrat.

Women occasionally suffer a diminution in their fecundating power, or become absolutely sterile after severe or mismanaged child-births—abortions, syphilis or gonorrhœa—or any other cause which may alter and impair the condition of the parts. What the precise alteration may be must constitute the subject of special examination in each particular case.

Dr. Davis observes that the condition of the male is of course to be investigated, and enumerates most of the many causes of impotence on his part. Perhaps we may mention two curious circumstances to which he makes allusion. The first is an observation of Hippocrates, that the Scythians suffered from palsy of the erectores penis, in consequence of too much riding. Whether the modern Arab or the Cossack is found to display a similar infirmity, we leave to others to determine. The second circumstance is connected with a peculiar fancy of the Hottentots, who are said to occasionally submit to the removal of one of their testes, in order to improve their powers of agility.

When considering the treatment of a case of sterility, it is, or it should be, the object of the practitioner to discover its cause. It may be an unruptured hymen, the remedy for which is a crucial incision—or contracted vagina, the narrowness of which may be removed by tents or by bougies—or preternatural septa or fræna, which admit of destruction by a simple operation. The uterus may be absent, or the vagina may be filled by preternatural growths, or the cavity and passage of the cervix may be obstructed. Our readers may be aware that Dr. Mackintosh, of Edinburgh, attributes the presence of amenorrhœa to the frequent occurrence of the latter state. Consistently with this opinion, he advises and practises the use of the bougie. Dr. Davis seems to hint, that he has oftener ventured to propose this method, than succeeded in prevailing on patients to submit to it.

Dr. Davis speaks highly of the advantages derived from the employment of the speculum. He adverts to retention of the menstrual secretion in the uterus—to absence of its cavity—to obliteration of the passage through the Fallopian tubes—and to other possible organic lesions of the uterus or its appendages.

"The influence of AGE on the faculty of reproduction has already been noticed. The susceptibility to conception is said to be most vigorous between the ages of eighteen and thirty. Very early marriages are observed in many cases not to be productive till after the lapse of a few years subsequently to their celebration, when they often become so. But late marriages, such for instance as we may suppose to be contracted, on the part of the female, between the ages of 35 and 45, are much less promising than very early ones; inasmuch as in the one case, the chances of issue improve with every year, whilst in the other they sustain a more than proportional diminution. The indications of treatment in both are in some respects founded on the same or very similar principles. They chiefly consist in the adoption of such measures as are known to be best calculated to promote and to sustain a sound state of the general health, and an accurate and well-balanced performance of the functional actions of the system."

Dr. Davis adverts to the superiority of country over that of town air, and makes the following observations on the subject.

"It would appear from the more recent population returns for England and Wales, that the most healthy districts of this country, at the present moment, in which, therefore, human life, on the average scale of the population, is protracted to the longest period, are, Cheshire, Flintshire, the Isle of Anglesea, Pembrokeshire, and Carmarthenshire. It has often been observed, that women who have had no children while residents in towns, have become immediately prolific upon going to live in the country. A respectable lady who has resided for many years in New South Wales, informed the author, a short time ago, that she had known many instances of females who had ceased to bear children in Europe, becoming the mothers of second batches of children subsequently to their emigration to Botany Bay; adding, that the fact was so notorious that before she left that country it was become the subject of current observation at Sidney."

The recommendations with respect to food and to exercise are too obvious to require more special reference. Dr. Davis adverts to the popular belief that some women are naturally so cold and coy, that their passions are scarcely susceptible of excitement, and their marriage is in consequence unfruitful. But such indifference to sexual pleasures is itself so unnatural a condition, that it probably depends, at least in the greater number of instances, on some peculiar affection of the organs, or moral agency of circumstances on the mind. He mentions the case of a noble duke, who, unfaithful in old age to his marriage bed, discovered, whilst engaged in the venereal act, that his younger paramour was amusing herself by blowing on a downy feather, in order to keep it afloat in the air. He supposes that such coldness was that of uncongenial ages, and concludes, in the luscious manner of Sir Peter Teazle, that there can be little community between January and May. In reading the occurrence we are forcibly reminded of the annoyance of Mr. Shandy, who, whilst occupied in the actual production of a "homunculus," was interrupted by the unconnubial remark, that he had totally neglected to wind up the clock.

It has long been noticed, and that by the admirers of the sex with regret, that the fop or the gay cavalier is often more successful in the court of Love than the man of sterling worth or profound acquirements. Perhaps the two cases related by Capuron and Pinel may serve to display the reason of the preference, afforded by female levity or instinct, to him of the spur or the sword.

"Consulted by a gentleman's wife on account of great absence of mind, and, in short, of total incompetency on the part of her husband to complete the sexual act which he had the inconsideration to commence, Peyrilhe advised her to make him drink a little more wine than usual immediately before going to bed. The treatment proved effectual. Capuron, p. 262. A parallel case is mentioned by Pinel, of a mathematician, whose connubial duties were disturbed and made of no effect by his constant habit on those occasions of employing his mind in the solution of mathematical problems. The same innocent stratagem is represented to have proved successful also in that case."

Dr. Davis would seem to complain of the inordinate virility of some husbands, and he recommends at least one ample bleeding to moderate their vigour. Whether the possessor of great venereal power will think it worth while to submit to the discipline, it is not for us to decide.

Dr. Davis concludes the subject of sterility by an enumeration of real or reputed antiphrodisiacs. Leaving them to those who are possessed of sufficient amplitude of faith, we will simply observe, that the means that improve and maintain the health are probably best adapted to strengthening the generative as well as the other functions of the body.

---

### X. Case of the Discharge of a Dead Fœtus from a Fistulous Opening near the Umbilicus.

A woman was received into the Cork-st. Fever Hospital in 1828, with considerable enlargement of the abdomen. Her history, as far as it could be learned, was, that eight years before she had been in labour, which, after continuing for two days, suddenly ceased, and the child, as she expressed herself, rose up into her stomach : no delivery followed. After remaining in bad health for about two years, she again experienced the symptoms of pregnancy, and gave birth to a child, which did not survive ; but the former child still remained in the cavity of the belly, and during its continuance there she bore three children, the last of whom lived. Ultimately a fistulous opening formed near the umbilicus, which was enlarged, and the original child removed; it was in a state of wonderful preservation, measured twenty-two inches in length, and had attached to it about two feet of the umbilical cord.

---

### XI. Of the Fœtus Breathing and Crying in Utero.

I was called up one night by an intelligent pupil in the hospital, who informed me, that a very strange sound was observed to come from a patient in labour, resembling exactly the whine of a child.

On going into the labour ward, I found the nurses and pupils surrounding a patient's couch with out-stretched necks, listening with greatest intensity and amazement; and on approaching within about six feet of the bed, I distinctly heard a low moaning whine, resembling the faint and painful cry of a delicate seven months child; this became more distinct the nearer I approached the patient, and there could be no doubt whatever, that it came from the abdomen of the woman on the couch, however produced. Still sceptical, I applied the stethoscope, when the fact was proved beyond a doubt, as not only the cry mentioned, but the laboured respiration of the fœtus was perfectly audible. A vaginal examination was instituted, and the head was found presenting, but high in the pelvis. The parts were only partially dilated, although the membranes were ruptured, and the waters had drained off shortly before. This woman was not delivered for four hours, and the above phenomena were observed by several of the pupils, up to the time of the child's birth. This patient's name was Morell, the date of her delivery the 2nd of December, 1830.

This case not only establishes a curious, we had almost said incredible fact, but in a medico-legal point of view, is of some importance, and shews in a striking manner the futility of some of the tests most depended on in child murder. — *Dr. Kennedy on Obstetric Auscultation.*

---

### XII. Differences between the Physiology of the Fœtal, and that of the Perfect Heart.

The highly-interesting experiments and observations of M. Merat (Dict. des Sciences Médicales, vol. v. p. 452) will, we conceive, account for this fact. They prove the comparatively more perfect inherent vitality of the heart, the more nearly the animal approaches the state of fœtal existence, and also its decreased dependance on the nervous system. From a number of experiments made by this gentleman on rabbits, the facts he arrived at were, that on the excision of the heart from the body in two animals, one 1 day old, and one 30 days old, the sensibility of the heart in the former continued for fourteen minutes, while that of the latter was only observed for one minute after its excision. He also found, that the gaping *(baillements)* of the heart in the first continued evident for twenty minutes, whilst in the last it continued only for one minute and a third. In addition to this, he observed, that the destruction of the lumbar portion of the spinal marrow, in the first days after birth, did not suffice to arrest the circulation, but that when twenty days or so had elapsed, this almost always arrested it.

The conclusions which we would draw from these interesting facts, are, that the heart's action in the fœtus, and of course the circulation, on the well being of which fœtal existence more immediately depends, are much less under the influence or more independent of the brain and nervous system than are those in the adult or child. And this would appear to be another of those wise and beneficent provisions in our original conformation, with so many of which the animal structure abounds, as we know how much more precarious would be the life of the young, were a weak system, such as its is, subject to the effect which an acute and susceptible nervous organization would impart. How much more frequently would nature, by so gifting it, have frustrated her intentions expressed in the divine law, ' increase and multiply,' were the circulation in them to be easily checked by the functions of the brain and nervous system being impaired? And even, with this provision, do we not too often observe infants destroyed by the pressure on the head during the process of parturition?—*Ibid.*

---

### XIII. Umbilical Hernia of the Gravid Uterus.

I met once with a very remarkable case of the latter description in a woman who had had a number of children; when in labour of her second child, hernia took place at the umbilicus, which gradually increased in extent with each child she carried, until at length the impregnated uterus made its way completely out of the abdomen, and became suspended over the pubis. I saw her at the expiration of the ninth month when carrying her twelfth child, when the pendulous tumour corresponded with that represented in *Plate IV. Fig.* 2.—*Ibid.*

---

### XIV. Use of Auscultation in the Case of Still-born Children.

The author was some time since informed by the highly-talented Dr. M'Intosh, of Edinburgh, that he also had been for some time in the habit of using the stethoscope, to detect the heart's action, in cases of still-born children, and with the happiest results; having by its assistance discovered the heart pulsating in cases, in which, after relying on the usual means, he had judged further endeavours to establish vitality as useless.—*Ibid.*

---

### XV. Curious Case of Apoplectiform Disease; Communicated by Dr. Fletcher, of Chesterfield.

A lady, aged about 55, who had for some time laboured under a disease of the heart, supposed to be hypertrophy of the left ventricle, was seized very suddenly on the 15th August, 1831, with an attack of apoplexy. She was totally insensible and speechless, with strong convulsions; the mouth was drawn to one side, and the breathing was stertorous. As soon as relief could be procured, she was bled very freely by a respectable surgeon in this town, and I saw her about half an hour afterwards. When a considerable quantity of blood had been taken the convulsions began to abate, and other alarming symptoms to be somewhat mitigated. Cold applications were used to the head, and stimulating injections were administered, but there was a total inability of swallowing, and therefore medicines by the mouth could not be given. In a few hours the power of speech and capability of swallowing gradually returned, and in a few days she was comparatively well. In the November following she had another similar attack, but she was then a considerable distance from home, and I did not see her. I understand that the treatment was very similar to what had been previously adopted, excepting that she was not bled with the lancet, but cupped on the temples. She recovered from this attack, and was again seized in a similarly violent manner in May, and in September, 1832, and again in Sept. of the present year. All these attacks were very similar to each other and very alarming, and nearly the same treatment was adopted in them all; but in the intervals she has had several which were less serious, though still attended with much derangement of her general health. It may be observed generally that during the violence of the attacks the pulse is oppressed, the heart beats sluggishly, and the pulsations can scarcely be felt, but after some blood has been drawn, the action of the heart gradually returns, and in some time becomes extremely violent, from the returning circulation, with prodigious thumping and beating, just as water when it has been pent up for some time, rushes through the opposing barrier with increased impetuosity. The violent attacks have commonly continued for three or four hours, and then sensibility, recollection, and the power of speech, &c. have gradually returned. Thus it appears that in little more than two years this lady has had five very serious attacks of fits which have all the appearance and character of apoplexy, independent of several threatenings or indications which apparently were averted by the means which were adopted. These were bleeding at the arm, cupping, leeches, blisters, &c. The blue pill was also given for a length of time in small doses, as a grain once or twice a day, but the bowels would not bear any stronger dose. The most effectual remedy, and indeed the only one on which we could place any reliance, was bleeding with the lancet. In the last attack, when the blood did not flow freely from the arm, a cupping glass was applied over the region of the heart, and in a very short time the stream from the arm became more free. This effect was observed two or three times, and I suppose may be accounted for on the supposition, that the external stimulus gives some relief to the oppressed heart, and thereby removes part of the impediment to a free circulation.

I believe that every remedy which has been suggested by way of precaution or prevention has been submitted to by the patient, excepting a seton in the left side, and to that she constantly makes a decided objection. In the intervals her health is tolerably good, and she is benefited by taking exercise in a small carriage, but she cannot walk far at a time, and going up any ascending ground is apt to produce an unpleasant palpitation of the heart attended with dyspnœa.

*Remarks.* While we fear that there is organic disease of the heart in the foregoing case, we suspect that the phenomena, in the attacks, appertain more to epilepsy or hysteria than to genuine apoplexy. The oppressed state of the circulation during the paroxysms, is unfavourable to the idea of apoplexy—and the absence of all subsequent paralytic symptoms tends to strengthen the opinion of its being hystero-epileptic. We would suggest that a grain of nitrate of silver be given every night for a fortnight—then a grain twice a day, for three weeks—and three grains a day for a fortnight. This plan will not endanger the skin, and may work some change in this mysterious affection.

---

### XVI. Case of Wound of the Gluteal Artery. By R. Carmichael, Esq.*

Many, if not most of us, remember the poetical description of the case of the leech-catcher, contained in the works of the late John Bell. That case was one of a terrible character, an incision

---

* Dublin Journ. Nov. 1833.

of two feet in length—eight pounds of coagulated blood removed from the sac —and a deluge of fresh blood, followed by a loud whizzing noise and apparent extinction of the patient's life, constituting its faithful and its horrid features. The anatomist, relying on the seeming exactness of his science, has ventured to doubt and to dispute the sober reality of John Bell's statements, and a strong imagination has been thought to have lent its vivid colouring to the dull and diminutive objects of nature. The sceptic may feed his favourite passion with the modest and unobtrusive circumstances of the following case.

A young gentleman, aged 17, received accidentally in the right hip a wound from a pen-knife, which penetrated as far as the handle would permit. This was instantly followed by a gush of blood so strong as to dash against the contiguous wall of the chamber. The hæmorrhage was immediately arrested by a medical man.

Three days afterwards the patient imprudently rose from his bed and walked down stairs. He had scarcely returned to his room when he felt an acute pain in the hip immediately succeeded by tumefaction. This daily increased, and on the 19th of September of the present year, eleven days after the occurrence of the accident, Mr. Carmichael was requested to visit him.

"On examination I found the entire right hip considerably swollen and firm to the feel, the skin was slightly discoloured, having somewhat the appearance that a bruise would present. The trochanter could scarcely be felt, so great was the tumefaction. On measuring the two hips, by passing a tape between the thighs to the anterior superior spinous process of the ileum of each, the affected hip measured two inches more than the sound one; the upper part of the thigh was also so much swollen, that its circumference measured more by an inch and a half than the other; the integuments were also discoloured more or less even to the ham. The small cicatrix of the wound was situated about half an inch above the presumed situation of the upper margin of the ischiatic notch, where the gluteal artery emerges from the pelvis. No pulsation was evident to the eye, even on the most minute examination, but the strong pulsation of an aneurismal tumor was manifested to the ear by either immediate or mediate auscultation."

Mr. Carmichael very reasonably supposed from the preceding circumstances that the case was one of diffused aneurism from wound. He resolved to offer the patient the chance that general means could afford. He directed the abstraction of ten ounces of blood from the arm, draughts containing tincture of digitalis were given every sixth hour, a cold lotion was applied to the tumefied parts, and absolute rest in the recumbent position enjoined. This plan, with occasional opiates to meet pain and uneasiness, was persevered in during five days, but no benefit was derived; on the contrary, the tumefaction of the hip and entire limb was obviously increasing, and the state of the patient was so distressing, that even he himself became anxious for the operation, which was performed on the 24th of September, in the presence of Messrs. Colles, Adams, M'Dowell, Hutton, Logan, and Doctor Brown. It would be difficult and unjust to abbreviate the already brief notes of this successful operation.

"The patient being placed upon a table, lying on his face, I commenced the operation by an incision five inches in length, commencing an inch below the superior posterior spinous process of the ileum, and about the same distance from the margin of the sacrum, and continued it in a line obliquely extending downwards to the trochanter major. The gluteus maximus and medius were then rapidly divided, or rather their fibres separated (as the incision ran in the direction of the fibres) to the same extent as that of the integuments. The coagulated blood forming the tumour then became apparent through the sac, or condensed cellular membrane with which it was covered. This was divided the whole extent of the incision by running a buttoned bistoury quickly along the finger introduced into the sac, and its contents, consisting of from one to two pounds of coagulated blood, were emptied rapidly out with both hands into a soup-plate, which it completely filled.—A large jet of fresh blood instantly filled the cavity I had emptied, but the precise spot whence it came being perceived, I was enabled by pressure with the finger to prevent any farther effusion, while that which had been just poured out was removed by the sponge. It was obviously the trunk of the gluteal artery just as it debouches from the ischiatic notch, which had been wounded. I endeavoured, but in vain, to secure the artery by means of the tenaculum. I had then recourse to a common needle of large size, and with this instrument was immediately successful in passing a ligature around the bleeding vessel, and of preventing all farther hemorrhage. After having waited some little time, to ascertain if the artery was perfectly secured, lint was introduced to the bottom of the wound, as it was not likely that union by the first intention would take place between the walls of the extensive cavity which contained the coagulated blood. The patient was then put to bed, and an anodyne given to him."

On the third day the external dressings were removed. On the fourth, the greater part of the lint contained in the cavity came away, followed by a flow of matter of good quality. On the sixth, the remainder of the lint and the ligature were discharged. The report is closed on the sixteenth day, when the patient is said to be completely convalescent, and the wound rapidly healing.

### XVII. Dr. Hart on the Fungating Venereal Ulcer.*

We have lately directed the attention of our readers to the complicated questions connected with syphilis and gonorrhœa. We promised, or we threatened, for our readers may be tempted, by the various character of their occupations or their tastes, to consider what we uttered as a promise or a threat, to revert to the subject on appropriate occasions.* Such an opportunity is presented to us now.

Dr. Hart, whose experience, he assures us, is considerable, has made some remarks on what he has denominated the fungating ulcer, which we cannot permit to pass without challenge. That experience becomes an additional reason for disseminating his opinions, if we think they are correct—for disputing them, if inclined to suspect that they are wrong. He commences by the remark, that this form of ulceration is distinguishable, which we grant, and should be distinguished, which we allow, from the genuine Hunterian chancre. But where is the Hunterian chancre to be found? Seldom or never in the Lock Hospital of London. We cannot avoid entertaining the suspicion, that the striking reference to the Hunterian chancre betrays a disposition to the theory which considers this the genuine syphilis. The subsequent portion of Dr. Hart's brief observations may tend to confirm this natural idea. In order that our readers may fully understand the nature of the sores to which Dr. Hart refers, we transcribe his description without abbreviation.

"This form of disease commences in one or more vesicles, seated on the outer or inner surface of the prepuce, on the cervix, more rarely on the glans or corona glandis. In females, it mostly occurs in the recess between the labia and nymphæ, on the inner surface of the latter, at the posterior commissure, and sometimes at the verge of the anus.

* Dublin Journ. No. XI.

* This is no idle flourish of words. We were gravely told by an able and an eminent *physician*, that our long review of the work of Mr. Wallace would infallibly lead to the destruction of this Journal. But our melancholy anticipations were in some degree corrected, by the reception of a letter from a distinguished *surgeon*, thanking us warmly for the labour we had expended, and the sentiments we had expressed. Probably, the analysis of a work on cholera would have called forth precisely opposite opinions.

Each vesicle, after a few days, is succeeded by an ulcer, which presents the following characters, a well-defined sharp edge, with an elevated border, when on the prepuce, the surface of the ulcer is generally concave, and covered with a yellow, or greenish-yellow coating of tenacious pus: often there is a profuse discharge of pus, more especially if the ulcer be on the inner surface of the prepuce, or at the cervix: the pus, in this case, is mostly cream-coloured, and of uniform consistence. This form of ulcer is not so frequently solitary as the Hunterian chancre, but generally occurs in a crop consisting of two or more.

There is generally a good deal of pain accompanying this affection. The inguinal glands sometimes become tender and enlarged, but scarcely ever suppurate.

When this ulcer is neglected or improperly treated, an exuberant granulation sprouts from its surface, which is hard and firm when its seat is the glans, and softer when it occurs on the prepuce. I have seen this excrescence generally larger, softer, and of a paler colour, on the genitals of females than on those of males.

When the fungus is allowed to continue for any length of time, it acquires a greater degree of hardness, and is more difficult of removal; it often expands, so as that its edge overlaps the skin around the margin of the ulcer."

We recognize the sore, and admit the general truth of the description. But the eye of the experienced observer of syphilis detects, without difficulty, many omissions. In the angle of the prepuce and the glans, this sore sometimes commences as an extensive vesication, rather than as solitary vesicles. It often produces phymosis. At the orifice of a prepuce naturally narrow, it often occasions a peculiarly radiated appearance, but the gentle attempt to retract the skin discovers the small and circular yellow ulcerations. The sore on the prepuce constantly inoculates the glans. On the inferior surface of the penis, the sores from the lodgement of discharge become confluent, and the opposite scrotum is infected, whilst the scrotum, in its turn, becomes the means of inoculating the contiguous thigh, and more especially its junction with the perinæum.

Successive crops of these sores appear, until the patient is completely cured. This is a frequent and obvious occurrence.

On the labia of the woman, they are sometimes seen descending like a string of beads from the upper to the under commissure. This condition is evidently the result of the natural approximation of the parts, and the contagious property of the discharge.

We might notice other omissions of our author, but we pass to a very important consideration—the liability, or otherwise, to secondary symptoms.

"I have not known a single instance where this ulcer was followed by secondary symptoms, and I therefore consider it to be a purely local affection. I have had frequent opportunities of ascertaining that it was contagious. Men under my treatment for this affection frequently communicated it to their wives, in whom it invariably exhibited exactly the same appearances as those above described."

The proverbial difference of doctors is amply displayed in the present instance. Dr. Hart declares that he has never witnessed secondary symptoms, and the word of a gentleman is always sacred. But we own that we *have* witnessed secondary symptoms, and that so often and so clearly, that we now invariably treat the case with the view to this liability. It is singular that at one time we entertained the same opinion as our author, and only gave it up on witnessing a marked case of secondary eruption. The patient was in the Lock Hospital of London—the sores were in the angle, and precisely such as Dr. Hart has described. They were healed by the red wash and applications of the nitrate of silver, combined with active purgation. The patient was dismissed apparently cured, but returned in a short time with the true syphilitic psoriasis and lepra. Since the occurrence of that case we have seen too many of a similar character. A patient was lately discharged from the institution, who underwent a course of inunction for the actual combination of these sores, with the small pustular eruption. We will not multiply examples. Were we called on we could furnish the details. We need not inform Dr. Hart that one genuine case of secondary symptoms from such a sore, must induce the cautious and experienced surgeon to adopt the means of prevention in all cases.

These remarks will form an apt introduction to the consideration of the treatment.

" Mercury, given internally, is not only unnecessary but totally useless in this disease, which I have often seen it continue, not altered in the least, although the patient had been fully salivated. It is to be treated altogether by the application of escharotics. I have found nitrate of silver, applied in substance, the most effectual means of removing the excrescence, if soft, or preventing its formation if applied to the ulcer which precedes it. I have also used the sulphate of copper in substance, with advantage in such cases. It is sometimes, however, necessary, especially when the fungus acquires a considerable degree of hardness, and overlaps the surrounding skin, to excise the growth with a knife or pair of sharp scissors : but if the excision be not followed up by the application of the solid nitrate of silver or sulphate of copper to the surface exposed by the removal of the fungus, the latter will be reproduced in three or four days.

I have found some persons so timid as to refuse submitting to excision, or even to the application of caustic. In such cases I have recommended strong solutions of the above-mentioned escharotic substances, and the frequent use of a powder composed of savine and sal ammoniac, in equal parts, or the acetic acid as recommended by Mr. Carmichael. This treatment has in a few instances been successful, after having been persevered in for a sufficient length of time; but it has more frequently failed, and the mode of treatment originally objected to has been at last submitted to, and with its usual good effects in removing the disease."

Philosophers have been confounded, and the man of the world has scoffed, at the glaring contradictions of medical evidence. We shall not attempt the unprofitable task of endeavouring to reconcile conflicting statements. We must confess that we totally differ from our author, and we boldly affirm that we have neither found mercury unnecessary nor useless. Were the question pointedly preferred, we would feel compelled to reply that we have found it absolutely necessary and useful.

We could not so flatly oppose an observer whose accuracy of description we have candidly admitted without a particular reference to facts. Four months ago a gentleman applied to us with a marked and severe specimen of the sore or sores. We purged him actively for two or three days, subdued and removed the inflammatory action that usually accompanies these doses in the first instance, and then prescribed the blue-pill. The ulcers immediately began to heal, and then, but not before, we touched them lightly with the lunar caustic. The sores were cicatrized in about a fortnight, but the mercury was continued, with the aid of sarsaparilla, for the space of three weeks or a month. All induration was removed, and the gentleman has since been free from complaint. In the hospital books are the notes of numerous cases of this nature, treated successfully by mercury. Many display the staggering fact, that the ulcers could not be cured without it.

We freely acknowledge the excellent effects of the nitrate of silver. It is always useful, sometimes it may be regarded as almost indispensable. The best time for its employment is just when granulation has appeared. The best *local* treatment at first is emollient applications, or perhaps the black-wash —when the yellow and *spongy* surface is appearing the camphorated red-wash of Bates—and soon afterwards the use of the nitrate of silver. Excision we never saw required and we certainly condemn. Mercury supersedes the necessity for steel.

The best *general* treatment is, purging with calomel at night and senna in the morning, till all pyrexia and every inflammatory appearance is removed—then blue-pill, say three grains, twice daily, with an occasional dose of infusion of roses and salts—and finally after ten days or a fortnight, the combination of the blue-pill with the sarsaparilla. This treatment is simple, decisive, and successful. We speak from facts. Did we not we should be equally arrogant and unjustifiable.

---

### XVIII. CASE OF BONY UNION OF A FRACTURE OF THE NECK OF THE FEMUR, WITHIN THE CAPSULE. By E. STANLEY, Esq.*

A young man in his eighteenth year fell from the top of a loaded cart upon his right hip, the injury of which was attended by the following symptoms. He was wholly unable to move the limb, and suffered severe pain when it was moved by another person. The thigh was bent to a right angle with the pelvis, and could not by any means be extended. Abduction of the thigh was difficult. The limb was everted, at first slightly, afterwards in a greater degree. The soft parts around the hip joint were considerably swollen. There was no shortening of the limb, but rather the appearance of a lengthening of it in the erect posture, probably from the obliquity in the position of the pelvis. No crepitus could be felt in any movement of the limb.

The general opinion of the surgeons to whose judgment the case was submitted, pronounced it to be one of probable dislocation into the foramen ovale. Forcible extension was made by the pulleys, and the thigh was then moved in several directions to replace the head of the bone in its socket. The success which resulted from these manipulations is not specified by Mr. Stanley.

Two months after the accident the patient was admitted into St. Bartholomew's Hospital with symptoms of general ill-health. One month after his admission he was seized with an eruption, pronounced to be small-pox, and in two days afterwards he died.

In the examination of the body, no other morbid appearances were discovered besides those of the injured hip-joint. The capsule of the joint was entire, but a little thickened. The ligamentum teres was uninjured. A line of fracture extended obliquely through the neck of the femur, and entirely within the capsule. The neck of the bone was shortened, and its head, in consequence, approximated to the trochanter major. The fractured surfaces were in the closest apposition, and finally united nearly in their whole extent by bone. There was an irregular deposition of bone upon the neck of the femur, beneath its synovial and periosteal covering along the line of the fracture.

Mr. Stanley fairly observes upon the case:—

"It will be remarked that in the instance now recorded, notwithstanding the free and repeated examinations of the limb, and the forcible extension of it by the pulleys, in short, with every circumstance except the age of the patient, unfavourable for a bony union of the fracture, this had been nearly completed. If this case had occurred at an advanced period of life, we may be certain that there would have been but a very imperfect union of the fracture, and it shews satisfactorily, that in the ordinary cases of fracture of the neck of the femur within the capsule, the age of the patient and consequent deficiency of vascular action, especially in the separated head of the bone, is the most influential of the causes to which the failure of a bony union has been in general ascribed."

---

### XIX. DR. PATTERSON ON THE EFFECTS OF MAMMARY IRRITATION IN AMENORRHŒA.*

The sympathy between the uterus and

* Med. Chir. Trans. Vol. 18, Part I.

* Dublin Journal, No. XI.

mamma is familiar to practitioners, but their attention has been usually directed to the alterations produced in the condition of the latter, by the changes that occur in the state and in the functions of the former. The following facts would appear to prove that the influence of the one upon the other is reciprocal, and that the physician in acting upon the mamma can exert some degree of power on the uterus.

*Case* 1. Mary Reardon, æt. 24 years, of moderately corpulent habit, was admitted into the Rathkeale Hospital on the 10th of August, 1832. She laboured under slight synochial fever, which in a few days yielded to venesection and purgatives. On the 19th Aug. symptoms which were considered of a hysterical character presented themselves, with pain in the upper and outer part of the right side of the chest. For the latter affection a small sinapism was prescribed, but from inattention of the nurse, it was made so large that it covered a considerable portion of the mamma. The sinapism remained on for half an hour.

At the visit on the following morning the 20th August, Reardon complained that the right breast was exceedingly painful, the pain being very different in its character from that which she had before experienced. On examination, the whole side of the chest was found considerably swollen; there was slight diffused redness of the skin; and though the mamma itself was enlarged to four or five times its natural bulk, yet there was no circumscribed hardness, nor any tendency to suppurative inflammation.

On the 21st August, the right mamma and adjoining parts of the chest were found much more enlarged than they had been at the preceding visit. The left mamma and side of the thorax were unaffected, and it was announced by the nurse, that the catamenia had that morning appeared, and were then present in considerable quantity.

This discharge, which, as the patient stated, had been for two years and a half wholly suppressed, continued to flow for two days; then it began to decline, and with it the tumefaction of the mamma gradually disappeared.

The attention of Dr. Patterson was arrested by the agency apparently exerted by the sinapism placed upon the mamma, over the catamenial secretion. He tried the same means in the next case that was presented.

*Case* 2. Catherine Power, æt. 19, applied to Dr. Patterson, on the 14th Sept. 1832, complaining of headache, languor, loss of appetite, and inability to attend to her usual business, that of a servant. She stated that about the middle of April, the menstrual discharge being then present, she incatiously exposed herself to cold in washing clothes at a river. The catamenia then suddenly ceased, had not since returned, and from that period she had been constantly subject to ill-health. She had consulted different medical gentlemen, and taken a great variety of medicine with little advantage.

Dr. Patterson directed that the clavicular half of the right mamma should be covered with a sinapism. The consequence was that the whole right breast became much swollen, hot, and painful. The next morning the enlargement of the mamma was very much increased, the tumefaction having extended to the clavicle and axilla of the irritated side. There was no hard circumscribed or prominent tumor, but a painful diffuse elastic distention of the mammary gland and surrounding cellular substance. On that evening the catamenia appeared. They continued for two or three days, and in a week the girl was so well that Dr. Patterson discontinued his attendance.

Both patients have since continued to menstruate with regularity.

Dr. Patterson remarks with judgment and with candour, that it must not be supposed that mammary irritation is applicable to every form of amenorrhœa. He does more than admit the possibility of failure, he presents an instance. In order that the evidence may be laid before our readers, and that Dr. Patterson's laudable and uncommon candour may be fraught with as extensive benefit as he could wish, we shall adduce the unsuccessful as well as the favourable cases.

*Case* 3. Mary Fitzgibbon, æt. about 21 years, of spare habit, was affected with headach, and irregular dyspeptic symptoms. The headache permanent, with occasional aggravation; countenance and tongue chlorotic; mammæ undeveloped. The menses had been scanty and irregular from the 16th to the 19th year of her age, but during the last two years they have been totally suppressed. No apparent organic impediment.

A sinapism was first applied to one breast, and afterwards a similar application was made to both breasts at the same time. But though the sinapisms produced their ordinary effects, considerable pain and cutaneous irritation, yet the enlargement of the mammæ was very trifling, and there was no consequent uterine action.

---

### XX. CASE OF ŒSOPHAGOTOMY. By Mr. ARNOTT.

Mr. Arnott has been tempted to relate this case, because he can only discover the record of the operation having been three times performed upon the Continent, and so far as he knows it has never been done in this country.

*Case.* On the 22d December, Mr. Arnott was summoned to the Middlesex Hospital to see a boy two years and a quarter old. Six days previously he had " swallowed " a portion of the thick end of a rib of mutton, which had stuck in his throat, and for the removal of which ineffectual attempts had been resorted to. The child had been unable to swallow any thing but fluids; in other respects he seemed to suffer little.

" On introducing the finger to the utmost extent, deep below the entrance of the glottis and on the right side, a piece of bone could just be touched, projecting upwards. I endeavoured to unfix it but it was too low. Gullet forceps and Weiss's urethra forceps were tried, but could not be applied so as to seize it. A hook attached to a piece of whalebone, and another of strong wire were ineffectually endeavoured to be passed beyond it. An emetic was given which was followed by severe straining, (vomiting did not take place, probably from there being nothing in the stomach:) but the position of the bone was unaltered. Lastly, I applied gentle pressure with the tip of the finger on its point, but it did not undergo the least displacement."

Mr. Arnott expressed a wish that the child should be brought into the hospital. The father, however, refused his consent. For a fortnight, no additional suffering was experienced, but emaciation took place. On the 16th of January the child was brought to the hospital again. For the last few days his breathing had become occasionally oppressed, more especially at night. Mr. Arnott could still feel the bone with his finger, but his colleagues could not. Probably the finger of Mr. Arnott is a long one. In consultation, the operation of œsophagotomy was decided on. The steps of the operation were these.

" The child being laid on its back upon a pillow and the head turned a little to the left, an incision was made on the right side of the neck in the sulcus between the sterno-mastoid muscle on the outer, and the larynx and trachea on the inner side. It was commenced opposite to the upper part of the thyroid cartilage, and carried downwards about an inch and three quarters in length. In the subcutaneous cellular substance two vessels which bled were tied. Beneath the fascia, the omo-hyoideus muscle presented itself, running diagonally across the wound; it was readily pulled to the inner side, and the division of the cellular substance continued; the knife being directed inwards upon the edge of the larynx and trachea, (so as to avoid the carotid artery,) until the outer part of the sterno-thyroid muscle was exposed. The further separation of the parts was effected by the handle of the scalpel and the fingers. Two vessels which resisted the traction on the cellular substance, and ran laterally into the right lobe of the thyroid gland, which now started into view, had a ligature put round them by way of precaution. By means of a blunt hook, the gland was drawn inwards, and the larynx turned a little round on its axis, but the finger applied behind the lower part of this did not distinguish the bone. A male silver catheter was now introduced by the mouth, and its point made to project through the wound, carrying the dilateable gullet upon it. Into this, a small incision was made, and a pair of polypus forceps being inserted, the blades were expanded, and the wound easily dilated in a perpendicular direction, so as to admit the finger. With this, the bone was felt about half an inch lower down than the aperture, and the forceps being re-inserted it was laid hold of, disengaged, and extracted; and proved to be the spinous process of one of the lower dorsal vertebræ of a sheep."

Little blood was lost. The wound was not united by the first intention.

For some hours after the operation, the breathing was somewhat interrupted by mucus collecting in the throat, about the entrance of the glottis; but this being expelled, partly by the mouth, and partly by the wound, he had a good night. He had been fed by means of an elastic gum catheter passed through the mouth; but the following morning some difficulty occurred in carrying it down, and as it was judged imprudent to urge it, lest ulceration of the œsophagus had taken place, and pressure might be injuriously applied, it was introduced through the wound, which was easily accomplished, and was the method adhered to. This day was passed tranquilly; but in the course of the second night, the child's breathing was laborious, and on the morning of the succeeding day, was accompanied by wheezing: he was at the same time severely purged. In the afternoon, the difficulty of breathing increased, the countenance became anxious and slightly livid, and death occurred at 9, p.m. fifty-six hours subsequent to the operation.

*Dissection.* Slight redness at the under part of the pharynx. Two points of superficial ulceration in the upper part, and on opposite sides of the œsophagus. The opening made in the gullet by the operation was one half in the pharynx, and the other in the œsophagus, or rather, the under half was below the lower margin of the cricoid cartilage. There was no appearance of suppuration between the pharynx and œsophagus, and the anterior surface of the cervical vertebræ. The entrance to the glottis and the cavity of the larynx presented their natural appearance, as well as the upper part of the trachea; but the under part of this tube and the bronchi were inflamed. The right lung, with the exception of its upper part, was hepatized, and portions of it thrown into water, sunk. Sections of it presented a mottled gray appearance, and granulated texture, with here and there a drop of yellow matter from the extremity of the bronchial tubes. In a less degree and more partially, hepatization was observed in the left lung.

Mr. Arnott makes some sensible remarks upon the case. Their gist may briefly be said to be this:—First, that the projection of the foreign body externally is not necessary to indicate, nor is its absence calculated to dissuade from the operation (in this case there was no such projection);—secondly, that the operation may be proper, and even necessary, though deglutition is not totally impeded, and though suffocation is not threatened;—thirdly, that a danger hitherto unnoticed may follow the residence of a foreign body in the œsophagus, we allude to inflammation of the lung, and, consequently, that an early operation is advisable in order to avert this risk;—fourthly, that although the situation of the external incision must be regulated by that of the body to be removed, the termination of the pharynx, in the narrower œsophagus, is the part most likely to have occasioned the obstruction, and that certain precautions may be specified in the performance of the operation in this place. Those precautions may be most appropriately mentioned in the words of Mr. Arnott.

"In performing the operation, the situation of the external incision will, in some measure depend upon that of the body to be removed, but as the pharynx, tapering gradually in its descent, terminates in the œsophagus, immediately under the larynx, it is here that a bulky substance is most apt to be detained. In reaching the œsophagus at this place, taking as a centre a spot corresponding to the level of the lower margin of the cricoid cartilage and the first ring of the trachea, the only parts of consequence whose injury is to be dreaded are the inferior thyroideal artery and recurrent nerve, (the superior thyroideal artery being too high to run any risk;) but these will not be wounded, if the same plan is adopted as that in the case I have related, of separating the deeper-seated parts by the handle of the scalpel and the finger, instead of by the knife.—Here, they were not seen during the operation, in fact, they were not within the sphere of the wound, for on examining the parts after death, the artery and nerve in question were found below, and on the inner side of it. Still I am satisfied by trials on the dead body that the artery is likely to be divided, if the operation is completed by the knife, and hence, the expediency of proceeding deliberately, cutting but little at a time, sponging carefully, so as to see and avoid the artery, if possible, or to tie it immediately when cut. The recurrent nerve runs less risk, as it reaches the side of the trachea to which it is attached in its ascent, lower down. I do not allude to the carotid artery as being exposed to any peril. I think with Mr. Allan Burns, that 'he must be wanton indeed in the use of his knife, who hurts this vessel.'"

Mr. Arnott adverts to some other circumstances—the dilatation of the wound in the œsophagus by the forceps, an excellent suggestion of Sir C. Bell—the nourishment of the patient after the operation by clysters, and the introduction of an elastic gum tube into the stomach, from the mouth or from the wound. In a note, he refers to two cases lately published in the Journal Hebdomadaire, in which the operation was successfully performed. In one this was done on the eleventh, and in one on the eighth day after the accident. The patients were adults. The incisions were made on the left side of the neck.

The profession must feel indebted to Mr. Arnott, for the manner in which he has drawn attention to the subject.

---

### XXI. Cases of deep-seated Nævi treated by the Seton. By Mr. Macilwain.

This will conclude our present notices of the lately-published volume of the Medico-chirurgical Transactions. An account of the remaining papers will be found in our next number, and perhaps it will be found that they are few and comparatively unimportant. They consist of some remarks on the discharge of fatty matters from the bowels by Mr. Lloyd and Dr. Elliotson—of a paper on irritation of the spinal cord and its nerves, in connexion with disease of the kidneys, by Mr. Stanley—and of cases of malignant tumors, by Mr. Langstaff and by Dr. Sims. These we may safely and satisfactorily postpone.

To return to the cases immediately before us. Mr. Fawdington, two or three years ago, published some cases of nævi treated by setons, which were noticed at the time in a number of this Journal. As the reference is not of any consequence, we need not take the trouble to make it more particular. Prior to the publication of Mr. Fawdington's practice, though not antecedent to its adoption, Mr. Macilwain had used the seton in the following case.

*Case* 1. In August, 1829, Mr. Macilwain was requested, in consultation with Mr. Wilson, of Northampton Square, to visit a child three months old, with a small tumor on the left side of the neck, a little below the angle of the jaw, supposed by that gentleman to be glandular. It had first been noticed three weeks after birth, and was then of about the dimensions of a pea. When seen by Mr. Macilwain, it was nearly the size of a small walnut, soft, elastic, smooth, compressible. It had many of the characters of a suppurating gland, but differing from it in some respects, Mr. Macilwain suspected its nature, and suspecting, made a more particular examination.

"I remarked that the softness was not exactly that conveyed by suppuration; it wanted something which I scarcely know how to describe, shall I say the central fluidity of suppuration? The tumour seemed universally soft and elastic, it was also so compressible that it appeared as if pressure, for the moment, reduced its volume; on looking very closely at its surface, just at the most projecting point, exceedingly minute vascular ramifications were discoverable. It was neither painful nor tender."

Not exactly knowing what the tumor was, he directed the application of a linseed poultice, an emollient substance, in which the doubts of surgeons and physicians may be not unfrequently observed to be immersed. After a fortnight the tumor was unchanged, and the cataplasm resolving neither it nor the uncertainty, a piece of soap-plaster was substituted for it.

"About a week, however, subsequent to this period, Mr. Wilson thought that he had discovered a pulsation in the swelling, and I was requested to see the patient again. I found that the tumour had increased in size, that the pulsation, though faint, was unequivocal, that the vascular ramifications before noticed had become more perceptible, and that when the child cried an impulse was distinctly conveyed to the tumour. These circumstances confirmed my growing suspicion that it was a deep-seated nævus, or vascular tumour. I accordingly directed the application of cold, by means of the common freezing mixture and subsequently by ice. Under the latter application there was at first a very sensible diminution in the bulk of the tumour, but afterwards the ice appeared to provoke a reaction followed by an augmentation of its volume."

Mr. Macilwain now met Mr. Stanley and Mr. Wilson in consultation. They agreed to endeavour to excite inflammation in the tumor by passing red-hot needles through its substance. The hot treatment succeeded no better than the cold, and a seton was resorted to by way of an experiment. The extent of the tumor was this. It reached above as high as the lobulus of the ear, which indeed with the contiguous portion of the auricle, it had considerably elevated from its usual situation; it extended below to within a half an inch of the clavicle, forwards it occupied the cheek as far as the anterior edge of the masseter muscle, and posteriorly it reached to within about half an inch of the mastoid process. A considerable plexus of vessels had become developed on its surface, and towards the ear a deepish redness was remarked, shewing the extreme vascularity of the tumor.

On the 5th December, 1829, Mr. M. passed a long circular needle, armed with two double threads of the silk commonly used for setons through the substance of the tumor from one side of its circumference at its base to the opposite; having adjusted the silk, so that it should as nearly as he could contrive it, fully occupy the space described by the transit of the needle. The structure seemed to yield before the instrument, but still to resist its puncture, so that he had the greatest possible difficulty in carrying its point through the mass, the needle bending so as to endanger its breaking, and yet by the sensation it imparted, it appeared to be pressing against a soft elastic matter. One jet of arterial blood followed the seton, just as when the simple punctures were employed. The constitutional disturbance excited by the seton did not subside for several days. The tumor increased with considerable rapidity.

On the 30th Mr. M. passed a second seton, with similar difficulty and similar immediate effects. The tumor was now of its greatest size. Its diameter downwards from above the lobulus of the ear was rather more than eight inches, from its anterior edge towards the mastoid process rather less; its circumference at the base was about seventeen inches. This, it must be owned, was a formidable tumor.

Suppuration soon became established in the line of both setons, and whenever a suppression of discharge took place the child became restless and uneasy. The tumor gradually diminished in bulk till May, 1832, when the setons were both thrown off in one night. There remained nothing more than a slight discolouration of the skin, and the cicatrices of the setons.

Sir Astley Cooper and other gentlemen saw the case during its progress, and entertained no doubt of the nature of the malady.

*Case* 2. In the Winter of 1830-1, Mr. Jacob requested our author's opinion of a tumor in the neck of a child, of the same age as the former patient.

It had been first perceived about a month after birth, and was then of the size of a horsebean; it was seated on the cheek immediately in front of the ear. When seen by Mr. Macilwain, in company with Mr. Jacob, the tumor had acquired the size of a duck's egg, and a number of small veins were seen upon the surface giving it a blueish aspect. The tumor was so similar to that which was described in the preceding case, that our author describes only the circumstances of difference. Its circumference was larger than in the first-mentioned case, its projection not quite so considerable, its situation in every respect alike, except that its anterior boundary came farther forward on the cheek; it had a somewhat more slippery feel, and though very compressible, did not yield quite so easily.

Mr. M. recommended the seton, and shortly afterwards Mr. Jacob introduced two. Mr. Jacob leaving town, the case was consigned to Mr. Macilwain, and the latter gentleman being also compelled to retire for a period to the country, the patient fell under the care of Mr. Lawrence at St. Bartholomew's Hospital. A dresser of that establishment removed the setons in about four months after their insertion. In rather less than a twelvemonth the mother again brought her child to Mr. Macilwain. The tumor had then only half the volume it exhibited when last he saw it. The mother declared that the diminution had not occurred *after* the withdrawal of the seton.

Mr. M. again introduced it. The jet of blood, and the great resistance to the needle, were precisely similar to those occurrences in the first mentioned case. The excitement occasioned by this seton, was much less considerable, and suppuration occurred after a shorter period. The diminution of the tumor has been so rapid, and the promise of its speedy dispersion is so flattering, that he has not thought it necessary to pass a second seton.

"On the present occasion I will only add, that the needle employed was about the size of that which is commonly used for working in worsted. In future I shall employ one a size or two larger, since there will be no danger of its breaking, whilst I have little doubt that, so far, the size of the instrument may be increased without danger of hæmorrhage."*

With the caution of experience Mr. Macilwain recommends discrimination and prudence in the employment of the seton, and anticipates that further modifications may be necessary. He concludes with a quotation not inappropriate to remedial means in every sense of their operation; for, happily, if they do not always effect the good that they may promise, neither do they always occasion the evil which they threaten.

Nec semper feriet, quodcunque minabitur arcus.

---

* "I would advise the surgeon not to neglect the plan of adjusting the quantity of the silk so that it should fully occupy the track made by the needle. In the present state of our knowledge on this subject, it would be at least imprudent to throw aside this precaution."

---

### XXII. Case of large Ovarian Tumor.

The following case of ovarian disease occurred in the practice of Mr. Marshall, an intelligent surgeon of Forfar, by whom it has been transmitted. We insert the particulars with pleasure.

"Margaret Emily, æt. 38, married, and the mother of several children, in 1828, supposing herself pregnant, was attacked with menorrhagia, in consequence of which a medical man saw her, who ascertained that she was not pregnant, but labouring under disease of the left ovarium. A tumor was distinctly felt in the left iliac region, movable from side to side by change of position. During three years she continued to suffer more or less from pain in the left side, with constitutional irritation, menorrhagia, hysterical affections, strangury, irregular bowels, anasarca with ascites, and latterly oppressed breathing. She menstruated regularly. The tumor progressively enlarged till, in 1831, the abdomen was so much distended as to render her condition very uncomfortable, and, in compliance with her urgent request, paracentesis was performed.

About eighteen pounds of fluid were removed, when the tumor was felt moving downwards and seen obstructing the opening. The trocar being again introduced, about six pounds of purulent matter were evacuated; the tumor descending farther the discharge stopped. The instrument was a third time introduced, but nothing following its removal, the patient was put to bed complaining of pain at the wound, which continued till her death, within 48 hours after the operation.

*Inspection.* A tumor was found occupying almost the whole abdominal cavity, resting upon the brim of the pelvis, and reaching to nearly the ensiform cartilage, which was distorted to one side evidently by pressure. The peritoneum, apparently inflamed in different parts, adhered intimately to the anterior and left lateral surfaces of the tumor, which consisted of a dark grey substance mixed with purulent matter, resembling very much a mass of tuberculated lung in progress of softening, easily broken down by handling, and unable to sustain its own weight. It was connected by an attachment about three inches in breadth, with the left broad ligament occupying the situation of the left ovarium, which had disappeared. At the lower and posterior part of the tumor a large cyst, containing a few ounces of pus, existed; this was the source of the purulent matter evacuated during the operation. The weight of this mass of disease, we calculated to be upwards of 40 pounds. No appearance of disease was observed in the uterus or right ovarium."

*Nov. 29th* 1833.

---

### XXIII. Ascites apparently cured, after Paracentesis Abdominis had been performed twelve Times.

The following fact, communicated by our friend Dr. Dickson, of Plymouth Hospital, is not undeserving of attention. The result can hardly be considered as determined; but so far as it has gone, it is highly satisfactory.

"Having lately had a case of ascites under my care, which has terminated in apparent recovery after the operation of paracentesis abdominis had been performed *twelve times*, it is presumed that the following brief notice of it will not be deemed undeserving of record.

When it is considered, out of the number of dropsical patients received into this hospital, how rarely the effusion is primary, and that it is usually consequent upon disease, generally far advanced, of one, and often more important organs, as the liver, spleen, kidneys, lungs, heart, &c. it necessarily follows that the operation of tapping seldom can be resorted to, with any expectation beyond that of its affording temporary relief. In the case of Lieutenant G——, R.N. aged 38, admitted with ascites on the 17th December last, there was little ground to authorize a more favourable conclusion; for there were evident enlargement and induration, both of the liver and spleen, the renal secretion was almost suspended, and the emaciation and debility were so considerable, that the measure in question was not adopted without my entertaining serious apprehensions of the result.

The operation, however, was borne better than was expected, and paracentesis abdominis was had recourse to twelve times, with increasing advantage, between the 29th of January and the 18th of July. The quantity of fluid abstracted, on each occasion except the last, by my friend Dr. Armstrong, being, upon an average, about twelve pints.

The medical treatment consisted chiefly of the frequent exhibition of hydragogue cathartics and the various diuretics, including the pyrola amulata, which, on many occasions, I have found to be very useful, but on others as inefficient—the internal and external use of mercury and of iodine, the diosma crenata, preparations of iron, and various other tonics, &c. But, as it would be impossible to give any analysis of a case which was under my care upwards of nine months, without entering into a long detail, suffice it to say, that his improvement latterly was so great, that when he was discharged for the benefit of change of air, on the 21st of September, he had scarcely required any medicine for several weeks; the kidneys were acting freely—the abdomen was reduced nearly to its natural size—the appetite was keen, and he was rapidly advancing in convalescence.

Dr. Good adduces a similar example from the *Common. Lit. Vrord*, 1735, of a person "cured after twelve operations;" and, in the present instance, it it is not unreasonable to anticipate an equally favourable result, if my late patient acts with prudence; for, after the lapse of more than two months, I yesterday learned that he continued to improve in health and strength, and, in fine, considered himself quite recovered.

D. T. H. DICKSON."

The concluding remarks of our excellent friend are valuable, but almost illegible. The printer's art can scarcely decipher and unfold the hieroglyphic characters.

"I have also met with two cases of empyema, which singularly enough occurred about the same time last year, (for Sir A. Cooper, who was here at the time, said he had met with but one such in his practice,) which were relieved by what might be termed a natural paracentesis thoracis; the one terminated fatally, the other, after being reduced to the last degree of emaciation by hectic fever, and the long-continued discharge, finally left the hospital quite convalescent, and even plump, after a sojourn of ten months.

I may also mention an extraordinary case of pericarditis seen by Sir William Burnett, in which the enormous quantity of five pints of purulent matter was found in the pericardium. The heart, though thickly coated with coagulable lymph, weighed only ten ounces. In another case of hypertrophia of the heart, this organ, when emptied of its blood, with the accreted pericardium, weighed three pounds nine ounces. A writer in the Edinburgh Journal, (No. XIX. p. 70,) mentions an enlarged heart, which weighed three pounds two ounces, and which he considered 'as large, if not larger than any as yet on record.' But he specifies it to have been 'Dutch weight,' which, if 24 ounces to the pound, if my recollection be correct, is of course a pound larger than the above—though notwithstanding you may think it worthy of notice. I have no time to go further to-night; but forgot to say, that in another case of ascites which terminated fatally, paracentesis was repeated ten times. Independently of the cases to which I have alluded, I believe the operation, generally speaking, is more successful in females than in males."

---

### XXIV. OFFICIAL REPORT OF CHOLERA IN PHILADELPHIA, 1832.

THE following extract from the Cholera Gazette of Philadelphia, is by no means out of place, even at this time.

"The regular westward progress of the great epidemic, known under the designation of the cholera, left but little doubt that its visitation would be extended to this continent. It became a subject of mingled curiosity and anxiety to watch the period of its arrival, and the point of its invasion.

In this state of uncertainty, intelligence arrived that the disease had appeared at Quebec on the 8th, and at Montreal on the 10th June, in both which cities it immediately assumed the character of a most destructive pestilence.

From the numbers of emigrants, who, about this period, had landed at Quebec, and arrived at Montreal from England and Ireland, a first impression was created, that they had been the means of transmitting the epidemic across the Atlantic. A more close investigation into the facts connected with the commencement of the disease in those cities, served to destroy this supposition. It could not be traced to importation. The emigrants and lower classes of the Canadians were attacked simultaneously in both cities. Numbers of the emigrants were in circumstances eminently predisposing them to suffer attacks of disease, and they and the lower Canadians were precisely the description of persons most obnoxious to the ravages of epidemic cholera, and such as have been universally observed to be its first victims.

The lines of communication between the cities of Quebec and Montreal, and the cities of the United States, are by the Richelieu river, Lake Champlain, and the northern canal leading to Troy and Albany; or by the St. Lawrence to Lake Ontario, to Buffalo, and by the Erie canal leading to Rochester and Albany. It was confidently expected that the disease would penetrate into the United States from Canada by these routes. Along the first, many cases of the disease did certainly occur in the persons of emigrants, but they terminated without its communication to others. On the contrary, the epidemic manifested a decided predilection for the shores of the St. Lawrence, successively attacking the towns and villages along its banks, then following the borders of Lake Ontario, until it entered Lake Erie.

While attention was directed to the northern and western boundary, supposed to be threatened by the invasion of the disease, it suddenly and most unexpectedly appeared in the city of New York.

The first case occurred, it is said, on the 24th June, when a man, a native citizen, residing at the corner of Gold and Frankfort streets, was attacked by the disease. Four cases soon succeeded, the location of which was in Cherry street. The subjects were Irish emigrants, who had arrived at Quebec in the autumn of 1831, and had resided in Albany until the month of May, when they removed to New York.

On the 27th June, the disease manifested itself in Bellevue Alms-house, distant about three miles from the city. The patient was an aged woman who had not left the house for three years, who had held no communication with the city, and no admission into the ward she occupied, had taken place for a month. Several cases immediately ensued in this and the other wards of the house. The epidemic reached its maximum in this establishment on the 11th July, and terminated on the 4th August.

In the city of New York, the climax of the epidemic arrived on the 21st of July, from which period it continued very steadily to decline.

The time that elapsed from the outbreaking of the epidemic at Quebec, and its appearance at New York, is a period of sixteen days, or nineteen at Bellevue Alms-house. The distance between the two cities in a direct line, is four hundred and fifty miles.

It is to be remarked, that all the intermediate cities on the sea-board of the province of New Brunswick and Nova Scotia; of the states of Maine, Massachusetts, and Rhode Island, remained entirely exempt from the epidemic; and even to the present period, except in Providence, Newport, and Boston, no cases have as yet appeared.

The moral resolution, calmness, and perfect freedom from alarm and panic, generally manifested by our citizens, and inspired by a thorough confidence in the efficacy of the preventive means enforced, in the advantages for salubrity of the city, and in its medical resources, contributed in no small degree to diminish the number of cases, and the intensity of the attacks. No stores were closed on account of the epidemic, and not more citizens left the city than usually abandon it every summer. A stranger entering our streets, from the busy throng and cheerful aspect of all he met, would never have suspected the existence of an unusual and a desolating scourge."

---

### XXV. RICEAN DOCTRINE OF CHOLERA.

It is a good many years ago that a worthy member of our profession (now dead) amused the world by a promulgation of what he termed the "Reecean Pandects of medicine." The Pandects did not last long, nor transmit the author's name to posterity; but they created a sensation—or a laugh—for the time; and that, perhaps, was all that the promulgator expected. A few years afterwards, when the cholera broke out epidemically in India, our brethren there were entertained with a "RICEAN PANDECT," issued by Dr. Tytler, and in which he undertook to trace that fatal malady to the use of damaged rice. Like the pandect of medicine at home, the rice doctrine had but a short existence in a tropical clime—and we were rather amused to find that the ingenious author had recently hit upon a plan of resuscitating his defunct doctrine before the London Medical Society. Considering that the cholera itself had nearly disappeared, and that the Society would be scarce of subjects, we have no doubt that Dr. Tytler brought forward the RICEAN PANDECT, as a mere joke, to make the Cockneys stare. While some appeared, on the prima facie statements of the Doctor to become converts, others remained sceptical—and a good number of malcontents evinced a slight degree of impatience at the joke, which they appeared to think was carried rather too far by the facetious wise man of the East. Dr. T. evinced very great ingenuity in his theories. Many people would have found some difficulty in accounting for the hop, step, and jump march of cholera—for its whimsical attachment to one bank of a river, or side of a street in preference to another bank or side—for its attacking the east wing of an army, or the larboard watch of a ship, although the soldiers and sailors were all using the same rice—for its passing through countries where little or no rice (and certainly none from India) was used—and for its first appearing at Sunderland, where there was no direct communication with India, instead of breaking out in London and Liverpool, where vessels from India, with plenty of rice, had been arriving almost daily, from 1817 to 1832. All these difficulties were easily overcome by the Doctor. He clearly explained how cholera desolated the cottage of the Highlander in the wilds of Rossshire, and the hovel of the Irishman, among the bogs of Allen—where oatmeal and potatoes were the common food, and where rice would not have been recognized if shewn to the inhabitants!

And how are all these difficulties got over—how is the *oryzean* etiology of cholera proved? He might have proved it by an argument, we think, the most irresistible—the most convincing. Did not every practitioner find that a cholera-patient voided *rice-water* stools before death? How could there be rice-water in the stools, if there were not rice in the food? This argument would have been demonstration itself! But the Doctor did not use it. The rock, as he expressed it, on which he stood, was the circumstance that, into a jail at Allahabad, which he guarded against bad rice, the cholera did not enter, though it prevailed in the town and country around. Why this is the very rock on which the contagionists have rested their chief arguments, ever since the epidemic began to spread westward. Dr. Tytler might find a whole chain or reef of these rocks, extending from the Ganges to the Clyde, and where cholera was excluded—not by keeping out bad rice, but by guarding against the introduction of contagion! Gateshead alone would carry conviction to the most decided oryzean theorist, that rice was not the cause of cholera. For nearly six weeks the disease prevailed at Newcastle upon Tyne, while the inhabitants of Gateshead, separated only by a bridge, remained free. But a North-east wind set in, and in 24 hours the disease was in 20 different parts of Gateshead!

But the Doctor is a perfect Proteus. No sooner do you jam him up in a corner than he transmogrifies himself into some other shape—and you have to begin a fresh attack! Thus, when the partiality of cholera for one bank of a river, was asked to be explained, the Doctor had an answer all cut and dry. The boats with bad grain all landed on one side, and those with good grain on the other! When it was asserted that some people got cholera who ate no rice at all—Never mind that, says the Doctor; they ate bread—and ground rice is frequently mixed with flour. When the Highlanders were instanced, who lived on oat-cake—the Doctor averred that his grandmother, or some other old woman, fed her children on ground rice! But some people did not eat bread at all, (as the poor Irish in the interior of Ireland)—and yet got cholera. No matter for that. Starch is frequently adulterated with rice, and being thus applied to the body by means of linen, is absorbed, and produces cholera. Some people, however, had not the luxury of starched shirts, and yet fell victims to the epidemic. What of that, says the Doctor,—Captain Vanslobberbreeks, of Batavia, assured me that rice taints the air—and thus we may get it through that medium, if we neither eat, drink, or wash our shirts!

In short, there has not been a more amusing exhibition at any medical society, in the annals of medicine, than this exposition of the Ricean doctrine. Nothing can be more clear than that Dr. Tytler is a sincere convert to his own theory, and that he has a firm belief in its truth. It is a mental delusion—for every individual has his "mentis gratissimus error"—before which all difficulties melt away—nay, where every opposing fact becomes only a buttress in support of the hallucination. Dr. Tytler's delusion is only a little more conspicuous than those of his neighbours. Each has his idola specus—and his is damaged rice. The universality of this agent is only equalled by that of the electric fluid itself. The pestilential ergot of rice—

> Lives through all life, extends through all extent;
> Spreads undivided, operates unspent!

We do not believe that the "three glorious *nights*" in Bolt-court have made a single convert to the doctrine—nor do we know any class of society who are likely to benefit by the oryzean discovery—except the MARKET-GARDENERS. We have no doubt that the magnates of this useful class of operatives will give Dr. Tytler a dinner at the Albion Tavern, in consequence of his having rescued fruit and vegetables from the stigma lately affixed to those delicious products of mother earth, as the cause of cholera.

---

### XXVI. BRISTOL MEDICAL SCHOOL.

We observe with pleasure that this great commercial city has added one more provincial school of medicine to the list. In a well-planned introductory lecture, at the opening of the school in October last, Mr. Hetling, the surgical lecturer, has pointed out, with much force, the benefits which must accrue from provincial schools of medicine. We can only make room for the following extract.

"The advantages resulting from these provincial medical schools must afford unmixed satisfaction to society in general; and in particular to parents, who will hail the introduction of them as a great domestic and moral improvement in their families, for it will be no longer necessary for you, or students in large cities, to be separated from the comforts and superior advantages of home, to reside for years in the cheerless, heartless, lodgings of London, where you would be exposed to so many more temptations likely to withdraw you from study.

It may not be misplaced here, again to recount the advantages this city possesses for a great medical establishment. With a population equal to that of many of the capitals of Europe, with a large and well regulated hospital and medical school, the student here may learn not only the principles and practice of Medicine and Surgery, but also observe and witness on a great scale the nature of most of the various diseases which afflict mankind in every part of the globe."

Dr. Carrick's address on opening the Bristol School, though hastily got up, having only 24 hours' notice, contains matter of much importance. We are not a little gratified to find the sentiments of this distinguished physician so completely in accordance with our own—sentiments which we have long endeavoured to impress on the minds of our professional brethren. The following passage will prove this.

"No man can be a thorough good Surgeon without being, as to education, a good Physician; nor can any man be a good and well qualified Physician without all the essentials of surgical education. I do not, however, consider the hitherto customary apprenticeship as at all an essential or necessary part of surgical education. On the contrary, an apprenticeship in a small town or city, where neither Infirmaries are to be found, nor Anatomical Lectures given, is a cruel waste of time. The benefit derivable from a five, or from a fifty years' apprenticeship, under such circumstances, is not worth three straws to its ostensible object, the apprentice. It may be otherwise, no doubt, in places where Medical Schools and Hospitals exist; as in such places some portion of the apprentice's time may be profitably employed in the lecture room, &c.; but this, be it observed, is altogether independent of the apprenticeship, and could be equally well accomplished without the apprenticeship as with it."

"I am not insensible to the very considerable improvement which has of late years taken place in the education and acquirements of the Apothecaries and general Practitioners of the present day, nor to the services which the Apothecaries' Company have rendered the profession and the public by their active legislative exertions, while the College of Physicians, the accredited guardians of the profession and the public; with whom these, and many other improvements ought to have originated, have actually done nothing, or nothing to the purpose; but sat dosing over their dignity, and exclusive privileges, while the wily and worshipful Company were cutting the grass under their feet.

In what I have stated above, I only meant to enforce a very obvious truism —that since Apothecaries and Surgeons act now as Physicians, the public have a right to expect that they should have the Physicians' education, and not the Druggists'; the first and most obvious step towards which would be, the abandonment of the time-killing apprenticeship. Without some such approximation in the education and qualification of all the members of the Profession, there never can exist amongst them that harmony and good will, and united energy and zeal so greatly to be wished, and not more important to their own interest than the public welfare."

We wish the Bristol Medical School every success.

---

XXVII. REMARKABLE CASE OF WOUNDED INTESTINE, WHICH OCCURRED IN THE PRACTICE OF J. D. DAVIDS, Surgeon, of Cowes.

[Extract of a Letter.]

*Case*. Dec. 11th, 1832. William Kemble, æt. 21, of spare habit, a butcher in Cowes, was gored in attempting to slaughter an ox without the precaution of making the animal fast. The accident happened two miles up the country, and he was brought to Cowes in a butcher's cart. I found him supported in a chair, vomiting, and in a state of great prostration. On removing his clothes I discovered about six inches of small intestine protruding through a wound in the lower part of the abdomen, just above Poupart's ligament, and about an inch external to the abdominal ring. I had him placed in bed, and on examining the intestine which was strangulated, I found that it had been completely perforated by the horn which entered it on one side and came out at the other, consequently making two apertures, through which I could pass my finger with ease. No fæces had escaped, nor had there apparently been much hemorrhage. The lips of the wounds were everted, exposing the mucous coat of the intestine. I immediately brought the larger wound together with two sutures, and the smaller with one, *and cut the ends of the silk close to the knots*. I then attempted to return the gut, but found that impracticable, without dilating the external wound, which I did with a probe pointed bistoury to the extent of about half an inch towards the ilium. By that means I was enabled to replace it in the abdomen. The external wound was closed with sutures supported by strips of adhesive plaster, and the patient was now (4 o'clock, p. m.) left. I saw him again at five, and found that he had vomited several times during my absence; there was also great tenderness to the touch generally, over the abdomen, and some reaction had taken place. Twelve leeches were applied to the abdomen. *Nine o'clock.* Pain much relieved by the loss of blood. I left him for the night, with an injunction that nothing should be given him but barley water.

12th. Had been restless and vomited several times during the night. There was a good deal of constitutional irritation, but no increase of pain in the abdomen. G. opii, gr. j. hac nocte.

13th. Had a quieter night and vomited much less frequently; complained of tenesmus. An enema was administered composed of ol. ricini, ℥iij. decoct. hordei, ℥v. with a view of emptying the rectum, which it did. Repet. pilula.

14th. Passed a tolerable night; vomited only once, but much annoyed with flatus. Enema repeated with the addition of infus. sennæ, ℥vj.; this brought away some fæces and grumous blood. Repet. pilula.

15th. Had another fair night; but complained of the bowels being painfully distended with flatus. Ol. ricini, ℥j. was given by the mouth in a little coffee and retained, which acted very satisfactorily on the bowels. Barley water had hitherto been his only sustenance; but to-day a little veal broth was allowed in addition. From this time, with the assistance of an opiate at night and an occasional aperient, he went on progressively mending till Christmas day, when he was induced to partake of some pheasant and mince pies for dinner. This indulgence was followed by excessive vomiting in the night of the 25th, and he was feverish and restless during the three succeeding days; however, attention to the bowels and abstemious diet, again brought him round, and he recovered from that period without the recurrence of any untoward symptom. A small abscess formed underneath the external wound, which discharged itself in due time, and the wound healed kindly by granulations. One of the sutures only made its way out through the wall of the abdomen; the other two I presume passed into the cavity of the intestine. There is a very considerable hernial tumor in the iliac region, rather above the seat of the wound, with, I conceive, adhesion of the intestine to the parietes of the abdomen, but it is attended with no inconvenience, as the man is able to undergo great fatigue, and is frequently to be seen riding saddleless on a rough trotting horse, with impunity. He wears a truss with a broad pad for security, and he assures me now, Oct. 1833, that his health is perfectly unimpaired by the injury.

---

### XXVIII. On the Use of the Stomach-Pump in Breaking Down Coagulated Blood in the Bladder. By J. Simpton, Surgeon, Ecclefechan, N. B.

*Case* 1. Mr. G—k, aged 76 years, has been subject to retention of urine for years past, which was generally relieved by fomentations, without the aid of the catheter. On the 18th February, 1826, I was requested to see him. He had passed no urine for 48 hours, the bladder was enormously distended, and upon introducing the catheter, which was done with some difficulty, owing to enlarged prostate and strong spasm of the neck of the bladder, a large quantity of brandy-coloured urine was drawn off. Inflammation succeeded, to subdue which, relays of leeches, fomentations, diluents, and in fact every antiphlogistic measure that the patient's age and constitution would admit of were found necessary. The distance from my own residence being three miles, and the bladder having now got so irritable as to render it imprudent, if not impossible, to leave a gum catheter introduced, my excellent assistant, Mr. Scott, (now surgeon, Pool Lane, Liverpool,) was obliged to remain to draw off the water, as it collected. Upon the inflammation subsiding blood began to come away in considerable quantities mixed with the urine. Things went on in this way for some days, when, on the 24th, a short time after the water had been drawn off, Mr. G. was seized with severe pain and the sense of distention, but on the instrument being re-introduced no water would flow. After some fruitless attempts Mr. Scott sent for me, when it appeared evident that coagula had formed, for, on withdrawing the catheter, its eyes were completely plugged, and on moving it from side to side when in the bladder, it gave a sensation to the fingers as if its point was immersed in some consistent or boggy substance, a particle of which I was unable to remove. The patient was in dreadful agony, and being made aware of the cause, begged me, for God's sake, "to cut in and take it out." Undisputed authority would have sanctioned a compliance with the old gentleman's request, for not long previous, and under nearly similar circumstances, Mr. Hutcheson,* with the approbation and in the presence of Sir Astley Cooper, cut in above the pubes, and scooped out the coagula with a table-spoon. Mr. H.'s patient died, and I had every reason to dread that mine would have gone the same road. Confident that the excruciating pain did not proceed from actual distention of the bladder, I resolved to break down the coagula by mechanical means, and for this purpose inserted a tube, connected with the shoulder of the stomach-pump, into a large double-eyed catheter, and threw in a quantity of tepid water with as much force as the pump would work, wriggling the catheter at the same time as much as possible, so that the streams might play in all directions. Pain was in some measure increased by distention, but this was of short duration, for in less than a minute after removing the tube the injected fluid came away by the catheter, mixed with a considerable quantity of blood. The bladder was completely emptied and immediate relief followed, so much so that the patient expressed himself "in Heaven." In a few hours blood again collected, coagulated, and was removed in the same way with the greatest ease.

The hæmorrhage now diminished, but it was the 9th of March before he could dispense with the use of the catheter, since which time he has not required its aid. Mr. G—k still lives.

The second case may be soon told. Mr. Thomas Maxwell, aged above 70, had been subject to retention of urine for some years, but was in the habit of using the catheter himself. The urine was often mixed with blood. On the 15th July, 1826, feeling more pain than usual, he introduced the instrument, but no water would flow. The pain becoming severe with sense of distention, I was requested to see him. From the report of the messenger I immediately suspected what was the matter, and dispatched the abovementioned Mr. Scott, prepared with catheter, pump, &c. Mr. S. found it a case, so far as coagula was concerned, exactly similar to the former, and in a few minutes relieved the poor man in a similar manner. He lived some years after, still, I believe, requiring the aid of the catheter, but without any more attacks of this kind.

Such cases as the above do not often occur, particularly to local practitioners, but, however seldom, it is certainly a desideratum to relieve the patient if possible without the use of the knife. Some die worn out with pain without the coagula being removed in any way.* Mr. Heavisides, an East Indian, was one. He entered the hospital with what was supposed retention of urine, but no water would flow by the catheter. He

* Vide Med. Chir. Review, for Jan. 1825, p. 224.

* Vide Medico-Chirurg. Review, for Oct. 1832, p. 492.

died next day, and a large coagula was found in the bladder. The bladder, to be sure, was diseased, but might not the man's life have been prolonged by washing out the mass? The presence of coagula, in my opinion, is easily detected, and by the above plan just as easily removed.

The injection tube belonging to the stomach-pump is what I use—its extremity is too large for the calibre of the catheter, but I connect them by a small brass tube, which slides over the point of the former and into the latter.

*Note by the Editor.* The syringe used by Mr. Costello and other surgeons, for injecting the bladder, after the lithotritic operation, will answer the purpose equally well as the stomach-pump.

---

### Medical Politics.

### XXIX. The Aldersgate Question.

This question, which was designated by some wiseacres as a local *squabble*, has turned out to be a very important subject of discussion, in general medical policy. Although the whole of the public is now acquainted with the resolutions adopted at the Freemason's Tavern, at the Westminster and London Medical Societies, and at various provincial meetings, on the subject of the Aldersgate Dispensary, we deem it essential to the welfare of our profession, to record one set of these resolutions in the pages of this Journal, as a specimen of all the others, confident that they will be read and referred to, long after the decease, not merely of the conductors, but of the youngest existing member of the profession. The following are the resolutions adopted at the Westminster Medical Society.

#### Westminster Medical Society.

At a meeting of the Members of this Society, held at the Hunterian Museum, on Saturday, October 26,

Mr. Pettigrew, President, in the chair,

It was moved by Dr. Gregory, seconded by Mr. Griffith, and resolved unanimously;

That in the opinion of this Society, the interest of the sick poor, and the respectability of the medical profession, equally require that the appointments to public charities should be free from the suspicion of being open to purchase.

Moved by Mr. Chinnock, seconded by Dr. Ryan, and resolved unanimously;

That in the opinion of this Society, the regulations lately adopted by the Governors of the General Dispensary, Aldersgate Street, permitting any person to attend, and vote personally, who should become a Governor, seven days prior to the election, amounts virtually to the sale of the professional appointments.

Moved by Dr. Webster, seconded by Mr. Millington, and resolved unanimously, with the exception of Dr. Epp;

That the cordial thanks of the Westminster Medical Society are due and are hereby given to Drs. Birkbeck, Clutterbuck, Lambe, and Roberts, and to Messrs. Salmon and Coulson, for their noble and disinterested conduct in resigning their offices rather than tacitly assent to the introduction of a law which compromises the honour and independence of the medical profession.

Moved by Dr. Copland, seconded by Dr. Sigmond, and resolved unanimously;

That the most respectful thanks of this Society be tendered to His Royal Highness the Duke of Sussex, for his liberal and enlightened conduct in retiring from the Presidency of the General Dispensary, thereby marking the sense His Royal Highness entertains of the conduct of the medical officers in resisting the adoption of a most obnoxious and pernicious regulation.

Moved by Dr. Somerville, seconded by Mr. Hunt, and resolved unanimously;

That in the opinion of this Society, any physician or surgeon who shall avail himself of such a law, and thus virtually purchase a professional appointment in any public charity, forfeits thereby his claim to the respect of his professional brethren.

Moved by Dr. Somerville, seconded by Dr. Sigmond, and resolved unanimously;

That the Society pledges itself to bring this amongst other grievances before such committee as may be appointed by the House of Commons to inquire into the practice and regulations of the medical profession.

Moved by Dr. Jewel, seconded by Dr. J. Wyat Crane, and resolved unanimously;

That the thanks of the Society are justly due, and are hereby tendered, to the medical practitioners of Sheffield, Cork, Nottingham, and other provincial towns, for their readiness to stand forward in support of the dignity of the medical profession.

Moved by Mr. Stodart, seconded by Mr. Greenwood, and resolved unanimously;

That these resolutions be signed by the Chairman, in behalf of the Society, and that they be inserted in the several medical journals, and following daily papers:—The Times, Herald, Chronicle, Globe, and Standard.

T. J. PETTIGREW, Chairman.
EDW. STODART, Secretary.

*Remarks.*—Posterity will hardly credit the statement, that, after such decided and unanimous sentiments of reprobation had been issued against the venal law of election, and against all who availed themselves of it, any member of the profession would have the meanness to bow his head to the petty tyrants of corruption, and, for the sake of a little malodorous praise from gin-shops, tallow-chandleries, and fishmongeries, forfeit the esteem, and incur the everlasting odium of his professional brethren! We are involuntarily induced to admire *courage*, whether moral or physical, even in the *worst cause;* but only when it is coupled with a belief that the possessor of that courage was actuated by some sense of honour and principle in the exhibition of it. It might almost be conjectured that a Burke, when he was strangling his victims, soothed his conscience with some faint idea that he was *promoting science;* but the nature of the placebo, which the PERDITI—the betrayers of their profession—can administer to their souls, is beyond our comprehension! We have racked our invention for a solution of this enigma—but in vain! In some minds, the love of *posthumous fame* is stronger than the love of living pelf or praise. Fame is of two kinds. A pillar stands in Rome to the memory of TRAJAN, the *good*—and another to that of PHOCAS, the *bad*. Trajan worked for FAME—Phocas for INFAMY. It may be said that *ambition* was the ruling passion, in both cases. Be it so! There is no accounting for tastes.—SATAN thought it—

"Better to rule in hell than serve in Heaven."

But how can we account for the preference to "*serve* in hell?" There is a hell in this world, as well as in the next. Every man's conscience is a hell, when he acts wrong—and if those who have plunged—or endeavoured to plunge a dagger in the heart of their profession, can drive from their own breasts the tortures of remorse, we heartily wish them all the advantages of an opiate on this occasion. But we are too well acquainted with human nature to believe that the false and fatal step which a few of our misguided brethren have taken, on this occasion, will prove other than an unfailing source of misery in their breasts to the latest day of their existence.

We understand, indeed, that they are endeavouring to console themselves and their friends with the reflection, that they are martyrs in a good cause—that they took the step they have done in order to break down an odious monopoly, or system of nepotism, that attaches itself to all our hospitals and dispensaries. There can be little doubt that more or less of this evil exists, and that it would be highly desirable that it should be remedied. But we are far from being sanguine in the hope of such purification, unless human nature should take a fit of reforming itself. This nepotism or private influence operates in every appointment, from the Crown down to the beadleship at the doors of our halls and churches. It influences promotion in the public service, and the selection of individuals for places of emolument in the law. This nepotism enters the sacred temples of our holy religion, and places mediocrity, too often, over merit. It puts a mitre on the head of INTEREST, and thereby keeps TALENT in a curacy for life! How can we expect that such a deep-rooted principle in human nature should fail to develop its activity in our hospitals and dispensaries! But what sort of remedy have the governors of the Aldersgate Dispensary proposed—and their medical helots accepted? They have adopted the system of PUBLIC AUCTION,—not as a substitute for nepotism, but as an additional or aggravating evil! The newly-revived venal law does not check, in the slightest degree, the old influence of nepotism. If a surgeon means to retire, he can easily desire his intended successor to make governors a few months before the secession, and thus avoid the pretended check of canvass which the governors, in their wisdom, have held forth as a panacea for nepotism.

If the governors had had the least desire or intention of really breaking down the medical monopoly, and not the expectation of enriching their treasury, they would have thrown open the competition, and permitted all regularly qualified physicians and surgeons of this metropolis to vote for such candidates as they considered best entitled to the professional charge of the institution. This would have insured an active competition, and what is more, it would, almost to a certainty, have placed merit at the head of the list. The sick poor would thus have been provided with the very best assistance —and no effort of private influence on the part of the existing medical officers could have prevented the fair exercise of the only voters qualified to judge of the capacities of the candidates. The subscription of a guinea, or of five guineas to a charity, could never have been designed originally to qualify non-professional subscribers for appreciating and deciding on professional merit. The statement which the Aldersgate mentors put forth respecting the power of the medical committee of the dispensary to decide on the pretensions of the candidates, is all a sophism, or rather a deception. They have no other power than that of seeing that the candidates have certain diplomas from certain bodies—which, everybody knows, is no criterion of fitness for an hospital or dispensary at all!

In fine, we sincerely trust that this incident will give origin to much improvement in our public institutions, and throughout the profession at large. But we cannot close our remarks, without adverting, with shame, to the conduct of our hospital and dispensary functionaries on this occasion. With three or four honorable exceptions, they all hung back, and took no part whatever with their brethren, in this contest with tyrannical governors and venal regulations.

---

### XXX. LIVERPOOL MEDICAL SOCIETY.

THE Liverpool Medical Society has met three times this present session in the Royal Institution Rooms. *Dr. Baird* had met with a most interesting and rather obscure case in private practice. An elderly gentleman of full habit was seized with a distressing urinary affection after some day or two of general indisposition. Urine at last dribbling away involuntarily. Pulse full, 108—(100) intermittent in one arm, which became cold—fingers blue and circulation ceased gradually from below upwards. Patient very restless—getting out and into bed continually. Sensation and motion but little impaired and consciousness unimpaired till the last. The pulse was imperceptible in the arm as high as the axilla. The extremity was quite cold, and got *perfectly black.* (See P.S.) Death. No *autopsy* procured. Query, What was the nature of the local affection? This case gave rise to much speculative discussion. Some thought the local disease was a form of Pott's gangrene. Others talked of ossification of inner coat of arteries—spiculæ being "perhaps" detached and plugging up the circulation, &c., others thought the case explained by Dupuytren's cases of inflammation of the veins, &c. But perhaps the most probable supposition (in the absence of proof which dissection would have afforded) was, that hæmorrhage had occurred into the sheath of the ganglionic system of nerves presiding over the irritability and contractility of the arteries of the limb: the pressure thence arising causing *apoplexy*, so to speak, of the extremity. Of course the rationalé of the local symptoms was based on Tiedemann and Gmelin's views, that the ganglia control and regulate the function of circulation.

The profession had a public meeting to-day, when resolutions were passed approving of the conduct of the late Aldergate Street Dispensary officers.

I cannot allow this opportunity to pass without mentioning symptoms which again and again I have known to occur when the nitrate of silver pill was taken at bed-time. Towards morning (pill being taken at bed-time) the patient frequently complained of an odd sensation as if slight *effervescence* were taking place, *here and there*, along the alimentary conduit. Headach, too, generally was present in the morning, in such instances.—*J. S. T.*

*P. S.* In Dr. Baird's case I should have mentioned that "the extremity became quite cold and perfectly black," and that the old gentleman felt as if a *wet* glove was on his arm, which he continually attempted to strip off with his other hand.

### XXXI. BRISTOL MEDICAL CONVERSATION SOCIETY.

THE first meeting of this Society took place at the Bristol Medical Library on the 19th of October last, when Dr. Davies presided, and delivered an address to the meeting. This event is likely to prove of general, rather than of local interest, and we hope that similar associations will take place in all our large towns. Dr. Davies took this opportunity of reporting two cases of strangulated hernia, as the commencement of the conversations.

The first case was that of a lady between 70 and 80 years of age, who had laboured under symptoms of strangulated hernia for more than forty-eight hours. The taxis having been unsuccessfully tried, Dr. Davies operated. The omentum was found to be much indurated; and a portion, weighing four ounces, was removed by the knife. Three ligatures were necessary. The old lady recovered perfectly.

*Case* 2. This was a young married woman, who, after one or two equivocal abortions, was suddenly seized with severe pain, nausea, vomiting, low, quick, and fluttering pulse, cold and clammy perspirations, &c. Dr. D. was led to suspect hernia; and, on examination, detected a femoral rupture. Under very disadvantageous circumstances, Dr. D. proceeded to the operation. The hernia was found to consist, anteriorly, of omentum, behind which, was a fold of intestine in a suspicious state of integrity. The omentum was indurated, in a great part, and removed by the knife, as it could not be returned with the intestine; and the patient did well, though she required very active treatment afterwards.

We conclude by again wishing prosperity to the Bristol Medical Conversation Society.

### XXXII. NAVAL MEDICAL OFFICERS.

*Admiralty-Office*, 15*th May*, 1833. The Right Honourable the Lords Commissioners of the Admiralty having been pleased to direct, "that no person be admitted to be a Candidate for the situation of Assistant Surgeon in the Royal Navy, who shall not produce a Certificate from one of the Royal Colleges of Surgeons of London, Edinburgh, and Dublin, of his fitness for that office; nor for that of Surgeon, unless he shall produce a Diploma, or Certificate, from one of the said Royal Colleges, founded on an examination to be passed subsequently to his appointment of Assistant Surgeon, as to the Candidate's fitness for the situation of Surgeon in the Navy; and that in every case the Candidate producing such Certificate or Diploma, shall also undergo a further examination before the Physician of the Navy, touching his qualifications in all the necessary branches and points of Medicine and Surgery for each of the steps in the Naval Medical Service;" The Physician of the Navy doth hereby signify, for the information of those persons to whom it may relate, that these regulations and directions will be strictly adhered to: and further, that previously to the admission of Assistant Surgeons into the Navy, it will be required that they produce proof of having received a classical education, and that they possess in particular a competent knowledge of Latin; also

That they have served an Apprenticeship, or have been employed in an Apothecary's shop for not less than two years.

That their Age be not less than 20 years, nor more than 26 years; and that they are unmarried.

That they have attended an Hospital in London, Edinburgh, Dublin, or Glasgow, for 12 Months.

That they have been engaged in actual dissections of the human body 12 Months; and

That they have attended Lectures, &c. on the following subjects, at established Schools of Eminence, for periods not less than hereunder stated; observing, however, that such Lectures will not be admitted for more than two different Branches of Science, by one Individual, viz.

| | Months. |
|---|---|
| Anatomy | 18 |
| Surgery | 18 |
| Theory of Medicine | 6 |
| Practice of ditto | 12 |
| Clinical Lectures on the Practice of Medicine & Surgery | 6 |
| Chemistry | 6 |
| Materia Medica | 6 |
| Midwifery | 6 |
| Botany | 6 |

Although the above are the only qualifications which are absolutely required in Candidates for the appointment of Assistant Surgeon, a preference will be given to those who, by possessing a knowledge of diseases of the Eye, and of any branch of science connected with the profession, such as Medical Jurisprudence, Natural History, Natural Philosophy, &c. appear to be more peculiarly eligible for admission into the Service.

It is also to be observed that, by the Rules of the Service, no Assistant Surgeon can be promoted to the Rank of Surgeon until he shall have served three years in the former capacity, one year of which must be in a Ship actually employed at Sea; and it is resolved that not any Diploma or Certificate of examination from either of the aforesaid Royal Colleges, shall be admitted towards the qualification for Surgeon, unless the Diploma or Certificate shall be obtained on an examination passed after a period of not less than three years from the date of the Party's admission into the Service; and whenever Assistant Surgeons already in the Service (whose professional Education may not be in accordance with the above) obtain leave to study previously to their passing for Surgeon, they will be required on their Examination to produce Testimonials of their having availed themselves of the period of leave to complete their Education agreeably to these Regulations. W. Burnett,

*Physician of the Navy.*

---

### XXXIII. Portrait of the late Jos. Brookes, F.R.S.

13, *Middlesex-place, Lisson Grove, Nov.* 16*th*, 1833.

Sir,—I am requested by the Committee formed for the purpose of devising the most efficient means of erecting a Monument or Memorial of the late Joshua Brookes, F.R.S., to announce, through the medium of your Review, their wish of its being generally understood, that an Engraving of the late Professor is published at One Guinea, the profits arising from the sale of which will be devoted to the above purpose, and subscriptions are also opened for the same object at the residence of each member of the Committee, and the Secretary, who respectfully solicit the aid of those members of the profession who may feel anxious to preserve some memorial of an eminently industrious and scientific anatomist.

I. C. Carpue, Esq. F.R.S. *Chairman.*
H. S. Chinnock, Esq. *Brompton.*
P. H. S. Colson, Esq. 25, *Goswell-road.*
Robt. Davey, Esq. 19, *Gt. Ormond-st.*
H. Davies, M.D. *Saville-street.*
T. Fowkes, Esq. *Mary-st. Regent's-pk.*
H. Hunt, Esq. 15, *Lower Brook-street.*
T. Hodges, Esq. *Grays-Inn lane.*
J. Johnson, M.D. *Suffolk-pl. Haymark.*
J. Kendrick, Esq. 12, *Manchester-street, Manchester-square.*
T. Litchfield, Esq. *Twickenham.*
J. Lavies, Esq. *King-street, Gt. George street, Westminster.*
J. Malyn, Esq. 25, *Duke-street, Westminster.*
J. Nicholles, Esq. 35, *Conduit-street.*
J. T. Pettigrew, Esq. F.R.S. *Saville-st.*

Trusting that you will find room for the insertion of this in your valuable Review, I am, Sir,

Your most obedient Servant,
H. Benham,
*Treasurer & Secretary.*

---

XXXIV. Obituary.

Died suddenly, at his residence in St. Paul's Churchyard, on the evening of the 1st of September, 1833, our oldest cotemporary—the father of the London medical press, in the 34th year of his age. Some of his intimate friends suspected that he was in a *decline* for some years past; but he himself never would allow of this—and maintained, up to the day of his death, that he was in perfect health—and that all his worldly affairs were in the most flourishing condition. We have not heard that any *post-mortem* examination has been made; but, as he was attended by two practitioners of talent and experience, we entertain no doubt that a report will be published of the cause of this awful and sudden visitation. We have learnt from very good authority, however, that the deceased evinced symptoms of ossification of the heart, with much embarrassment, both in the breathing and the *circulation,* for some time before his death.

Our late cotemporary first saw the light—if it deserved that name—in the dark purlieus of Red Lion-court, Fleet-street, on the first day of March, 1799—by the assistance of the late Dr. Bradley and Dr. Willich. Though apparently free from any hereditary taint, it was his good or bad fortune to be constantly in the hands of doctors—and it might be said, with strict truth, that he lived upon medicine—that his whole and sole food was physic! How he contrived to live so long, upon such innutritious provender, is the great wonder!

He had a very clever nurse, however, in the person of one Phillips, residing in Bridge-street, Blackfriars, who brought him up with great care. His first introduction to practice was greatly promoted by cow-pox, which had then been discovered, and the deceased acquired much fame and fortune by the part which he took in the discussions on that subject. His practice rapidly increased, so that in 1803, he had nearly *three thousand* names of patients on his books!

In 1805, a Scotch Doctor (Duncan) set up in opposition, and the deceased, in that year, saw a defalcation in his practice to the amount of seven or eight hundred patients. In 1810, another competitor came into the field; but did not affect the practice of the deceased in any material degree. In the following year, however, a company of apothecaries, among whom were Dr. Thomson and Dr. Burrows, started an opposition, and reduced the income of the deceased very alarmingly. In 1818, we first became acquainted with the deceased as a contemporary; and although he certainly treated us with great shyness and suspicion, we are not conscious of having ever injured him, or clandestinely deprived him of a single fee. Of late years, however, the number of competitors greatly accumulated, and the deceased, always of a sallow complexion, appeared to become affected with permanent jaundice. At length he suddenly expired—and thus fell the last of the Mohicans—or at least of the Monthlies—not an individual of that species now remaining in the British dominions!

The death of our patriarchal contemporary may teach the most youthful and the most robust of his surviving family to repress pride, and expect the same fate, sooner or later. No journalist ever kept the field so long as he who has just quitted this earthly scene. It was supposed that this very protracted existence, and the numerous progeny which he had all over the world, would have so securely propped him up, as to render him almost immortal. But journals, like their conductors, have their epochs of rise, acmé, and decline—a destiny which seems inseparable from every thing on the surface of our globe.

It is curious that our Parisian neighbours issue forth eight or ten monthly medical journals, while not one now exists in the United Kingdom.* Periodical medicine, in this country, has deserted the middle path—and finds no resting-place now between the hebdomadal and trimestral orbits. There are now five quarterly, and three weekly journals devoted to medical literature in the British Isles. Whose turn comes next to take leave of the stage? Each is, no doubt, inclined to hope that he will be spared a little longer than his neighbour.

"Et mihi forsan tibi quod negarit
Porriget hora!"

---

XXXV. Case of Amputation in an Infant seven weeks old. By J. Paul, M.D. Surgeon to Gray's Hospital, Elgin.

The following case is remarkable on account of the tender age of the patient. We can only spare space for the naked facts of the case.

"A male child, seven weeks old, was brought to me by his parents in the month of September, with an enormous swelling of the right leg, which had all the characteristic marks of fungus hæmatodes. The swelling was soft and elastic, bulged out in various parts, and presented a livid hue at the most depending point, with enlarged cutaneous veins. At birth two tumours were observable, the one running into the other; the lower one was said to be about the size of a turkey's egg, and the other somewhat less. The child's health was tolerably good.

The whole leg being involved in the morbid action of the disease, nothing but amputation above the knee even required the consideration of a moment, and the patient being so young this alternative was deemed almost too desperate; at least some delay was thought prudent. The parents were therefore advised to take their child home, and return when the swelling burst.

They had not been at home many days when the swelling did burst, and so profuse was the hemorrhage that in less than one minute the infant was in a state of syncope, and for two days life appeared to be almost extinct, so much were the vital endowments depressed. The hemorrhage was controlled by pressure, and the little patient rallied after a few days.

He was admitted into *Gray's Hospital* on the 3rd of October, and at this time, although not three weeks since I had seen him, it was manifest the disease was in progress. The swelling hung down from the malleolus internus on a line with the sole of the foot, and extended to the internal condyle of the femur. In the livid portion of the swelling ulceration had taken place, and a fungus, in appearance like brain, with portions of blood on its surface, had sprung up. It was two inches and a half in diameter, and the skin around its circumference appeared red and inflamed. The circumference of the limb across the fungus was eleven inches and a half, and nine and a half close to the knee. No part of the tibia could be discovered except close to the ankle-joint; the fibula could be traced, but there was an immense covering of dense and elastic cellular texture over it. The child looked pale, and the discharges from his bowels were profuse and of a greenish colour.

The following day, Oct. 4, with the assistance of Mr. William Robb and Mr. Robert Paterson, surgeons, and Mr.

---

* We forgot the Liverpool Monthly Gazette.

John Grigor, student in medicine, I amputated the limb above the knee, and used two lateral flaps. Scarcely a tablespoonful of blood was lost. About the same number of arteries as in the adult were secured, and whilst they were being secured the blood effused on the stump coagulated readily. The flaps were kept in apposition by means of stitches."

The child lived till the 2d of November, and then died of the consecutive fever, the stomach being entirely free from inflammation. We think Dr. Paul was justified, under the circumstances of the case, in performing the operation, and thus giving the little sufferer a chance, however small, of life.

---

### XXXVI. Remarkable Case of Pulmonary Abscess, unpreceded by any serious Symptoms.

[Communicated by J. A. Ore, Assist. Surg. 8th Royal Irish Hussars]

Thomas Glynn, aged 18. The subject of this case was one of those misguided people known in the wilds of Connaught by the designation of "Terry Alts, or "White-feet." This lad, along with several others, having been engaged in incendiary acts, was arrested in the night of the 19th March, 1831, and on being ordered to proceed along with the military escort to Gort, pleaded his inability to walk that distance, being six Irish miles; and was consequently left in his hut with a guard. On the following morning I was ordered to proceed and report on the state of his health and fitness to undertake the journey. On examination he was found to labour under *some difficulty of breathing*, attended with heat of surface and foul tongue; pulse about the natural standard, firm, and regular. He had a Burgundy-pitch plaster on his chest, recommended and supplied by *some St. John Long* in the neighbourhood. On interrogating him by means of a policeman, as my patient neither understood or spoke English, he stated that he never had any serious illness, or was prevented following his daily pursuits as an agricultural labourer, and there was much evidence that he had been engaged in many similar acts to that for which he was taken up, requiring personal energy and stratagem in their execution. Considering the very equivocal circumstances of his case, his transmission in a cart was determined upon, and I accompanied him to Gort. On his arrival there, he complained of fatigue, and that there might be no lack of humanity, he was taken into a vacant ward of the Detachment Hospital and a sentry placed over him; a few days afterwards he became an approver, and from this period the difficulty of breathing became mitigated, and was unaccompanied with the slight febrile symptoms which previously existed: there was no cough during the whole period he was under observation. On the morning of the 1st of April, he was somewhat agitated at the thoughts of being about to be confronted as a witness against his quondam friends, and having eat breakfast, set out in a post-chaise, accompanied by a party of Hussars, for Galway, where the Assizes were then being held. About four miles from Gort, the serjeant of the party, not perceiving him sitting up in the carriage, opened the door and found him dead. On examining the body to ascertain the cause of death, it was discovered to have proceeded from the rupture of an abscess in the right lung, containing no less than *eight pints* of a greenish-yellow pus: the left lung was perfectly sound, healthy in its structure, and of natural appearance. The viscera of the abdomen, pelvis, and head were more than usually healthy; the body was not emaciated; the external appearance or formation of the thorax gave no indication of such extensive disorganization as appeared on dissection. During the period he was under observation, he took merely some antimonial and purgative medicines.

The lung was removed quite entire, and after preparation forwarded to the Army Medical Museum at Chatham. This case is interesting as regards the enormous size of the abscess, unattended, from all that could be ascertained of the history of symptoms, by any indisposition of importance.

